# Enhanced Health Study Discoverability: Graph-Based Analysis Approach

**DOI:** 10.2196/86812

**Published:** 2026-08-18

**Authors:** Lea Gütebier, Stefan Groß, Benjamin Winter, Max Blumenstock, Martin Dugas, Volkmar Liebscher, Dagmar Waltemath, Ron Henkel

**Affiliations:** 1 Medical Informatics Laboratory, Institute for Community Medicine, Universitätsmedizin Greifswald, Walther-Rathenau-Straße 48, Greifswald, 17475, Germany, 49 3834 86 8374; 2 Department of Internal Medicine B/Cardiology, Universitätsmedizin Greifswald, Greifswald, Germany; 3 Partner Site North, DZHK (German Centre for Cardiovascular Research), Greifswald, Germany; 4 Institute of Medical Informatics, Heidelberg University, Heidelberg, Germany; 5 Institute of Mathematics and Computer Science, Universität Greifswald, Greifswald, Germany

**Keywords:** health studies, knowledge graph, health data integration, semantic interoperability, findability, accessibility, interoperability, and reusability, FAIR principles, study findability, data reuse

## Abstract

**Background:**

Efficiently finding and exploring relevant health studies is critical for informed, evidence-based health care. However, study information remains distributed across multiple resources, hindering interoperability, search, and reuse. Enhancing the findability of study data is a key challenge in promoting the findability, accessibility, interoperability, and reusability (FAIR) principles in health research.

**Objective:**

This study aimed to improve the findability and comparability of health studies by developing a semantically enriched graph-based framework that supports intuitive search and exploration for diverse stakeholders, including clinicians, researchers, and patients.

**Methods:**

We developed the BRAinS-Graph (“Biomedical Knowledge Graph for Recommending and Analysing Health Studies”), a semantically enriched knowledge base that integrates data from ClinicalTrials.gov, the Portal for Medical Data Models, the Unified Medical Language System, and MeSH into a single graph database. The framework applies an extract-transform-load process to integrate heterogeneous data structures and link related information across study resources and biomedical ontologies.

**Results:**

The BRAinS-Graph supports fine-grained, semantic searches across study metadata, eligibility criteria, and structural properties. Use cases illustrate its potential for clinicians, patients, and researchers, including analyses of study type distributions for meta-analyses and the identification of studies relevant to individual patients.

**Conclusions:**

By integrating heterogeneous study data into one interconnected knowledge base, the BRAinS-Graph improves the findability, accessibility, and reusability of study information. This work establishes a foundation for graph-based study recommendation systems and cross-institutional research infrastructures.

## Introduction

Informed clinical decision-making relies on the outcomes of evidence-based research studies, including clinical and epidemiological studies. This evidence is the basis for treatment options, determines the efficacy and effectiveness of drugs or vaccines, and ultimately improves patient care quality. Study data are intensely curated, complete, and of high quality and are therefore valuable assets for evidence-based health research. Digitalization, changes in legal regulations, and modern data integration pipelines allow researchers to reuse existing study data in wider contexts.

Finding suitable studies for secondary research and accessing study data for meta-analyses or pooled data analyses are important yet time-consuming and laborious tasks [1,2]. Patients should be able to find and select studies that are relevant to their individual needs; scientists have a high interest in reusing study data for their own research; and clinicians need to find the latest research findings to reevaluate their own treatment practices. The comparison of different studies helps design new study protocols, identify potential study participants, and place scientific results in the context of previous studies [2,3].

The research community at large has recognized the benefits of reusable data, as promoted by the findability, accessibility, interoperability, and reusability (FAIR) guiding principles for data stewardship [4], and making studies findable has become a key goal of study repositories, such as ClinicalTrials.gov [5]. The World Health Organization (WHO) emphasizes the importance of study registration, stating that “the registration of all interventional trials is a scientific, ethical, and moral responsibility” [6]. The registration of clinical studies in public repositories ensures transparency, accessibility, and ultimately reuse of study data. In fact, some medical journals require registration in a study repository as a precondition for publication [7]. This was recognized more than 20 years ago. However, we are still faced with the problem of study records being distributed across different repositories, leading to issues with interoperability, redundant data storage, and complicated data access [1]. Consequently, targeted searches for study data and metadata remain difficult and inefficient, challenging the ability to find, retrieve, and explore studies of interest.

To address these challenges, we developed a concept and prototype for an integrated knowledge base. This single point of access provides new possibilities for the search, exploration, and retrieval of clinical studies and thereby improves study findability and accessibility. The knowledge base is built on a graph database and provides a framework for the storage and integration of publicly available study resources and biomedical ontologies. The 4 study data sources are the international study repository ClinicalTrials.gov [5]; the Portal for Medical Data Models (MDM Portal) [8], a collection of medical data models; the Unified Medical Language System (UMLS) [9]; and the MeSH [10]. Complementary study metadata, medical data models, and ontologies are accessible within a single, semantically enriched knowledge graph. The main contributions of our work are as follows:

- Introducing a novel knowledge base for the search and exploration of medical studies and related study forms, enriching ClinicalTrials.gov data with structured eligibility criteria and ontological knowledge, thereby increasing the findability of studies.
- Providing example use cases to demonstrate the search and exploration possibilities of the knowledge base, showcasing how integrated ontologies can be used to query the linked study domains.
- Providing a publicly available, open-source implementation of the framework that can be reused for individual knowledge bases or other projects.

To the best of our knowledge, this is the first knowledge graph that integrates not only data from ClinicalTrials.gov but also complementary data, such as study forms from the MDM Portal, along with relevant ontologies [9,10].

## Methods

### Data Sources

**Figure 1.**
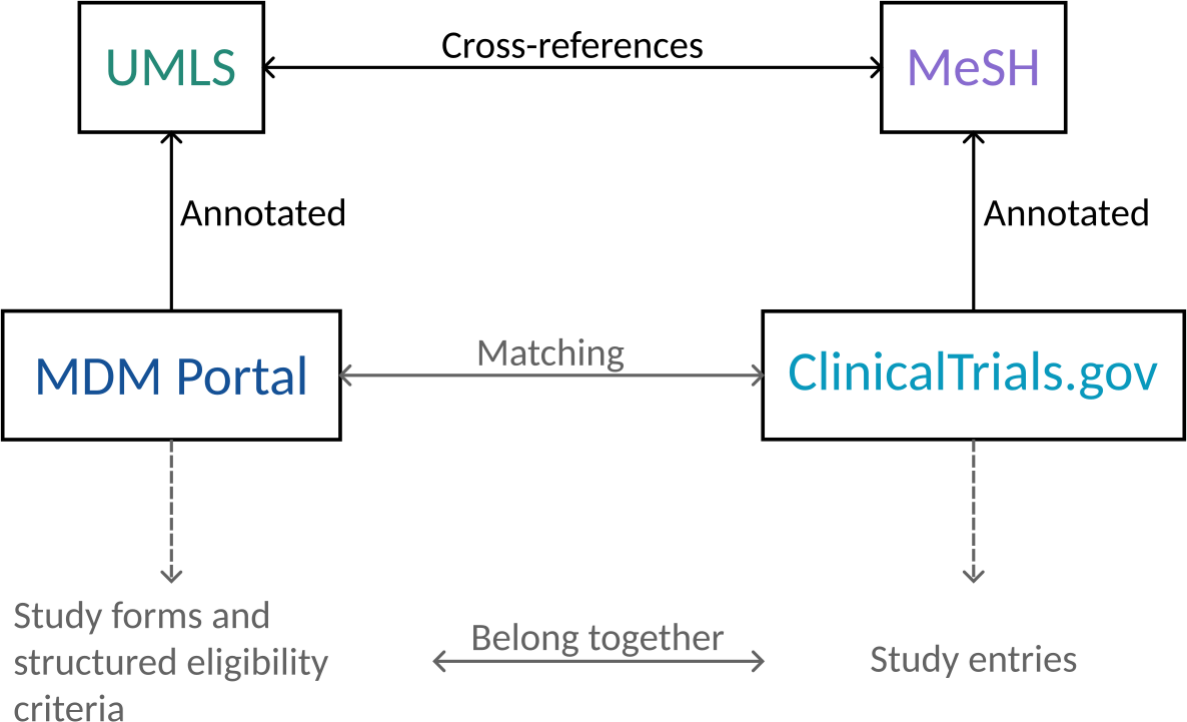
Overview of the 4 data sources and their interconnections. ClinicalTrials.gov provides study entries annotated with MeSH terms, while the Portal for Medical Data Models (the MDM Portal) provides medical data models, such as study forms and structured eligibility criteria, annotated with Unified Medical Language System (UMLS) concepts. MeSH and UMLS contain the referenced ontology terms, with cross-references linking concepts across the 2 ontologies.

The 4 data sources for the creation of the knowledge base are ClinicalTrials.gov [5], the MDM Portal [8], the UMLS [9], and MeSH [10]. Figure 1 provides an overview of the 4 resources and their interconnections. The selection of the data sources is based on the relative significance and impact of the respective resources. We chose ClinicalTrials.gov and the MDM Portal as the primary sources, as they meet these criteria.

#### Studies

ClinicalTrials.gov is one of the largest and most prominent study repositories, hosted by the National Library of Medicine. We interviewed several domain experts who highlighted it as the main repository for studies. Clinical research studies, including interventional and observational studies, are organized into study records that are associated with a set of data elements. Study entries comprise different sections with study data fields from the repository’s data dictionary [11]. Data can be downloaded in different formats, such as JSON, CSV, and XML. For integration into our knowledge base, we downloaded study entries as individual JSON files.

#### Medical Data Models

The MDM Portal contains structural metadata, called medical data models [8,12]. The MDM Portal has been established as an important system in medical research by providing a significant metadata repository. The portal manages and uses more than 25,000 medical data models, which are consistently stored in the XML-based Clinical Data Interchange Standards Consortium Operational Data Model (CDISC ODM) format [13] and are semantically annotated with UMLS terms, ensuring standardization and semantic encoding [14]. The provided medical data models include, among others, study data dictionaries, study base datasets, structured eligibility criteria for studies, and case report forms. ODM files can be downloaded directly from the MDM Portal after registration or via their API. API documentation is provided [15]. We downloaded individual metadata models from the MDM Portal in CDISC ODM format (version 1.3.2).

#### Unified Medical Language System

The UMLS combines health and biomedical vocabularies into a metathesaurus that links interrelated concepts within and across these vocabularies [9]. A concept consists of synonymous terms that describe biomedical information. These concepts are linked by relationships that are either specific to the UMLS or originate from the source vocabularies. A so-called Semantic Network is used to categorize concepts in the metathesaurus according to their semantic types [16], such as “Organism,” “Anatomical Structure,” and “Biological Function.” We used the API to retrieve UMLS concepts and their semantic relationships from the UMLS version 2025AA, level 0 subset, restricting the selection to concepts with English concept names and parent-child relationships.

#### MeSH Data Source

The MeSH is a biomedical and health-related vocabulary that was originally developed for the purpose of indexing publications. MeSH organizes terms hierarchically in a tree structure. We used the original source, thus downloading the MeSH 2025 vocabulary in the .*nt* file format directly from the website of the National Library of Medicine. It should be noted that MeSH does not include explicit cross-references to other ontologies, which we address during the mapping process using cross-references from the UMLS.

### Input Data

For demonstration purposes, this paper focuses on the context of diabetes. Diabetes was selected for its clinical importance, high public health relevance, and the availability of corresponding studies and medical data models in the selected resources. To populate the knowledge base, we imported 1760 medical data models from the MDM Portal and 1319 study records from ClinicalTrials.gov into the graph. Data were obtained by searching the MDM Portal for the keyword “diabetes” on February 18, 2025, and the ClinicalTrials.gov Expert Search by entering the Search Query “studies on diabetes in Germany” on February 19, 2025, without using additional filters. In ClinicalTrials.gov, the search results include multicountry studies, meaning that all studies with at least one study location in Germany are included in the dataset.

In addition, we integrated the MeSH vocabulary and 1,574,945 concepts from the UMLS. An overview of the data sources and record counts is provided in Table 1. The resulting knowledge base comprises approximately 3 million nodes and 8 million relationships.

**Table 1.** Overview of the dataset.

Data source and content	Count, n
ClinicalTrials.gov^a^	
Study entries	1319
MeSH annotations	4052
MDM Portal^a^	
Medical data models	1760
UMLS annotations	117,093
MeSH^b^	
MeSH terms	348,733
UMLS^b^	
UMLS concepts	1,574,945

a Contributes study-related entries and ontology annotations.

b Provides biomedical ontology terms and concepts.

### Graph Data Model

Graph databases represent data in a network that consists of nodes and edges, called a graph, offering a flexible and intuitive way of linking data. They have proven effective in systems biology [17] and systems biomedicine [18] and are becoming increasingly important in medicine and clinical applications [19]. In the medical domain, they serve as valuable tools for exploring and analyzing complex data and support AI-driven methods, such as graph-based machine learning [19]. ClinicalTrials.gov graphs exist [20], but they focus on COVID-19 studies [21-23]. Unlike relational databases, which require the mapping of heterogeneous data structures onto predefined schemas, graph databases allow the preservation of the original data structure and seamless integration with other types of data.

For our knowledge base, we chose one of the most widely used graph database systems, Neo4j. Neo4j is conceptually based on a labeled property graph [24], extending the graph by assigning labels to nodes, types to relationships, and adding node and relationship properties. Node labels represent the type of data stored (eg, label *Gene* or *Study*), whereas relationship types represent the type of connection between individual data points. Both nodes and relationships can have properties that provide more details about the stored data (eg, property study title).

The following paragraph explains the methodology of an extract-transform-load (ETL) process used to transfer data from the data sources to a labeled property graph.

### Framework for ETL Process

The construction of the knowledge base follows an ETL process, which involves the extraction, transformation, and loading of data into a graph database. The ETL process comprises the following steps. First, the data are extracted from their source repository. They are then transformed according to the graph data model. The model defines the representation of data as nodes and relationships in the graph. Finally, the transformed data are loaded into the graph database.

**Figure 2.**
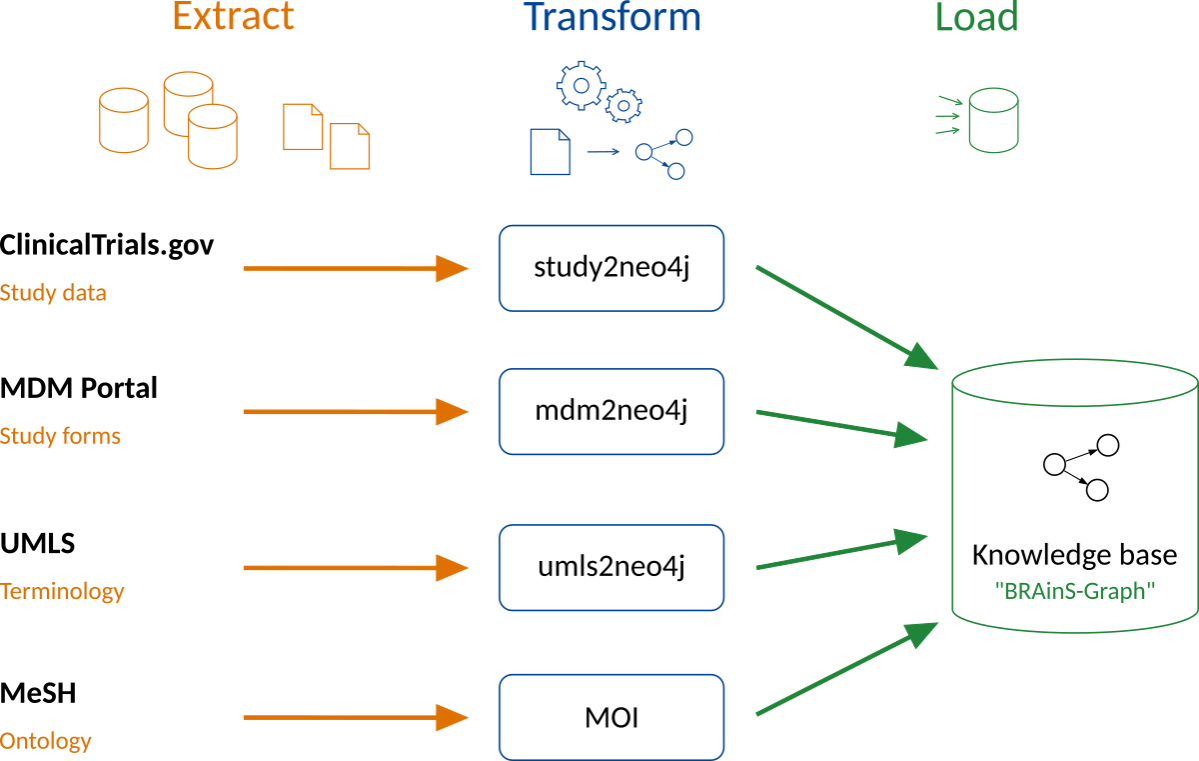
Overview of the BRAinS-Graph (Biomedical Knowledge Graph for Recommending and Analysing Health Studies) build process. The build process is an ETL process that includes extracting, transforming, and loading of data. Data are extracted from the 4 data sources. Then, an individual data loader for each data source transforms the data to a graph representation, which is then loaded into the graph database, thereby creating the BRAinS-Graph. MDM Portal: Portal for Medical Data Models; UMLS: Unified Medical Language System.

The framework for building the knowledge base is written in Python (version 3.11.2). It handles the connection to a Neo4j database (version 5.16.0), the ETL process for the source data, and the consecutive mapping of complementary data. An overview of the knowledge base build process is shown in Figure 2. Executing an ETL process in the framework first establishes a connection to an active Neo4j instance. The ETL process includes 4 data transformation modules, one for each data source (for a description of the data sources, see the *Results* section). Each module starts by extracting the contents from the files downloaded from each respective data source. The files are parsed, the file content is processed, and the extracted data are transformed into nodes and relationships in accordance with the specified data model, which is a labeled property graph. For example, data from the XML-based CDISC ODM files from the MDM Portal are structured into elements and attributes of an element. The transformation onto the labeled property graph model represents elements as nodes in the graph, which are hierarchically connected by relationships, and attributes as respective node properties. Consequently, this transformation specifies which data become nodes and what become node properties. At the end of each transformation module, the data are loaded into the Neo4j database.

### Build Process and User Guide

Our framework can be used to build the knowledge base on a user’s local machine. Once the user has downloaded the repository, they must (1) download and store the data source files and (2) specify the connection parameters for a running Neo4j instance in a configuration file. The data transformation modules for each data source can be executed either independently or in sequence. For more detailed instructions about the individual modules, we direct the reader to the README file provided in the public repository. The repository contains the framework and configuration setup, while users must obtain the data themselves under the respective licenses. We note that the method described here can be applied to different diseases. The procedure is described in the documentation of the code repository. The main steps requiring time and technical knowledge are obtaining the source data for import, while adhering to the respective licensing, and setting up a running Neo4j instance with the respective configurations (see the technical requirements in Multimedia Appendix 1).

The graph building framework is free, libre, and open-source software (FLOSS). It can therefore be reused in other projects while ensuring that future changes remain open source.

### Mapping of Data Sources

To integrate the various data sources, we mapped the data across the different study-related resources and implemented direct relationships between nodes to connect the respective data domains. Each study resource is represented as a subgraph, and these subgraphs are linked based on shared, complementary, and overlapping information, such as study identifiers, MeSH and UMLS terms, and cross-references between ontologies. We implemented the following types of relationships: (1) relationships between the so-called “study resources” (ClinicalTrials.gov and the MDM Portal), (2) relationships between the study resource annotations and their respective ontologies (ie, MeSH and UMLS), and (3) cross-references between integrated ontologies. For the first relationship type, we linked entries from the MDM Portal to those in ClinicalTrials.gov using the National Clinical Trial (NCT) identifier. Specifically, we checked the MDM subgraph for *Study* nodes whose Object Identifier (OID) matches an NCT identifier. If an MDM Study node’s OID property matched an NCT identifier of a ClinicalTrials.gov study node, we created a direct relationship between the 2 nodes. If no match was found with the OID property, we tried to match the Name property of the MDM *Study* node. If the name corresponded to a ClinicalTrials.gov NCT identifier, we analogously created a relationship between the nodes. We manually checked the results of the name-based matching for a random sample of studies.

The mapping of study resources and vocabularies is based on the resources’ annotations. For each annotation, we match the respective concept in the respective vocabulary based on the concept’s unique ontology identifier and create a relationship pointing from the annotation to the vocabulary concept. For example, we matched the concept names of the MDM study’s *Alias* nodes with the concept unique identifiers (CUIs) of the concepts in the UMLS. There is no duplication of concepts in the UMLS graph, meaning that if multiple MDM nodes are annotated with the same UMLS concept, the multiple *Alias* nodes will point to the same UMLS node. Analogously, we matched the MeSH terms from ClinicalTrials.gov annotations to their respective nodes in the integrated MeSH.

**Figure 3.**
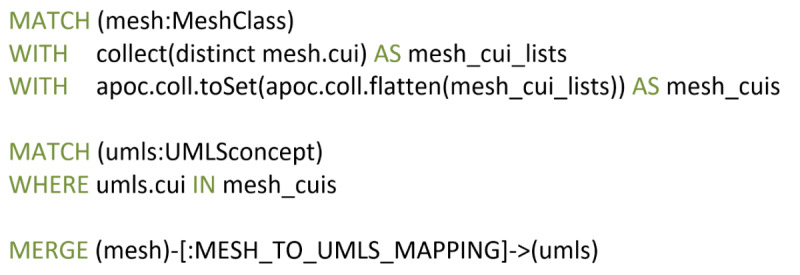
Post-processing step of mapping MeSH terms to Unified Medical Language System (UMLS) concepts. The query first matches all MeSH nodes and collects their distinct concept unique identifiers (CUIs) into a list, which is then converted into a set to remove duplicates. It then matches UMLS nodes whose CUI appears in this set. Finally, the query creates MESH TO UMLS MAPPING relationships from each MeSH node to the corresponding UMLS concepts. The MERGE clause ensures that relationships are created only if they do not already exist, preventing duplicates (listing is included in Multimedia Appendix 1).

Cross-references between the integrated vocabularies, that is, between MeSH and UMLS, are mapped based on the unique CUI identifier used in both MeSH and UMLS. Therefore, we create relationships between *MeshClass* nodes and *UMLSconcept* nodes for each node pair that has the same identifiers stored in their respective CUI property. We implemented all relationships based on these mappings in a post-processing step using Cypher queries (eg, Figure 3).

### Ethical Considerations

This work reused (meta)data records that were publicly available and retrieved from public databases. No primary data collection was conducted. No data from human participants or identifiable personal information were used. In accordance with data protection laws, ethical approval was not required for this work.

## Results

### Overview

Our knowledge base is built on a graph database, designed to improve the findability and exploration of studies by linking related study metadata and forms in one knowledge base. If data are dispersed and not easily findable, why not consolidate them? Here, we describe the selected data sources, present the design of the knowledge base, explain how study data are linked across data sources, and demonstrate how the resulting knowledge base enables exploration.

### The BRAinS-Graph Knowledge Base

#### Data Sources

We selected 4 data sources to create the knowledge base: ClinicalTrials.gov [5], which is a comprehensive and prominent international study repository; the MDM Portal, which is Europe’s largest collection of medical data models with semantically annotated elements [8]; the UMLS [9]; and MeSH [10]. Medical data models in the MDM Portal complement the study entries in ClinicalTrials.gov. For example, while ClinicalTrials.gov contains information about clinical studies conducted by the German health centers, such as the German Center for Diabetes Research and the German Center for Cardiovascular Research, the MDM Portal provides the corresponding base datasets. It also hosts data dictionaries for selected studies, such as the Study of Health in Pomerania [25]. A key advantage of the MDM Portal is that eligibility criteria are machine-readable and semantically annotated, unlike the free-text entries in ClinicalTrials.gov. However, the MDM Portal does not cover all studies registered in ClinicalTrials.gov. We included the UMLS and MeSH as additional data sources because the MDM Portal semantically annotates metadata models with UMLS terms, whereas ClinicalTrials.gov entries are annotated with MeSH terms.

#### Knowledge Base Design

Conventionally, relational databases are used for storing structured data. However, they are less suited for storing highly connected and heterogeneous data. While relational databases fall short of representing complex interconnections in a dataset, graph databases give equal importance to both data entities and the relationships between them [26]. In addition, their schema-optional nature makes them well suited for integrating different data formats, such as combining complementary study information in a shared knowledge base. The network structure of the integrated ontologies in particular makes a graph database a natural choice for their representation [27].

**Figure 4.**
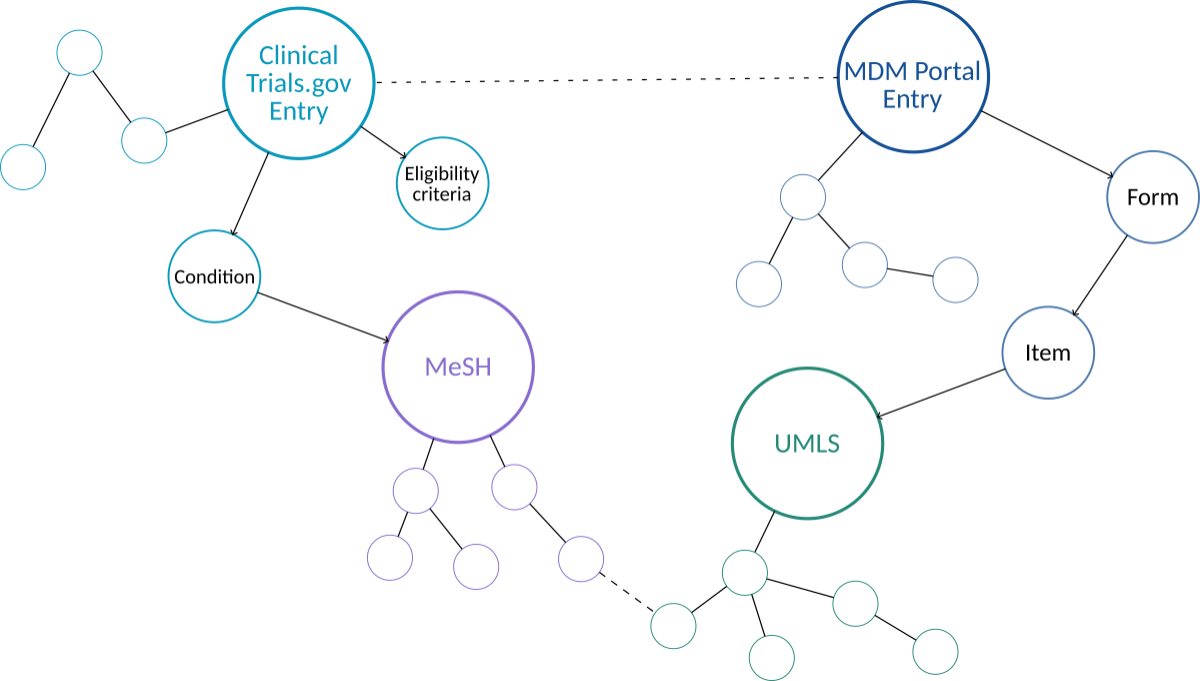
Metaview of the BRAinS-Graph (Biomedical Knowledge Graph for Recommending and Analysing Health Studies). This overview shows the graph model with the 4 entry points, which are labeled as ClinicalTrials.gov Entry, MDM Portal Entry, MeSH, and Unified Medical Language System (UMLS). Nodes are represented as circles, while relationships are depicted as arrows between circles. Dotted lines indicate domain-crossing relationships. The nodes are color-coded according to their data source, with turquoise indicating nodes belonging to the ClinicalTrials.gov subgraph, dark blue indicating nodes belonging to the MDM Portal subgraph, purple indicating MeSH nodes, and green indicating UMLS nodes.

The BRAinS-Graph (“Biomedical Knowledge Graph for Recommending and Analysing Health Studies”) is a labeled property graph, in which data are represented as nodes and relationships. Both nodes and relationships can have properties. Additionally, nodes have labels (see the *Methods* section for details). We analyzed each of the 4 data sources individually and created a corresponding graph representation, analogously to our previously published framework, mdm2neo4j, for medical data models from the MDM Portal [28]. Each graph has a designated central node that serves as an “entry point” to the respective data. The current implementation includes 117 distinct node labels and 125 types of relationships. An overview of the graph model with the 4 entry points is shown in Figure 4. For a more detailed graph model, see Figures S1 and S2 in Multimedia Appendix 1.

Here, we summarize the main node labels and relationships of the graph model. Note that node labels are written in italics in this paper to distinguish them from the actual data (eg, the node label UMLSconcept represents a UMLS concept). The 4 main node labels for the data sources are ClinicalTrialsEntry (for ClinicalTrials.gov), Study (for the MDM Portal), MeshClass (for MeSH), and UMLSconcept (for UMLS).

The ClinicalTrialsEntry node is the central node of a study from ClinicalTrials.gov. It has 2 outgoing relationships that represent information about the study. One relationship connects the ClinicalTrialsEntry node to a protocolSection node, which connects to further subcategory nodes representing different study modules. These include a contactsLocationsModule node (connected to locations and geoPoint nodes), a sponsorCollaboratorsModule node (connected to leadSponsor and collaborators nodes), and a designModule node (connected to phase nodes), thus representing the structure of a ClinicalTrials.gov entry. Additionally, the ClinicalTrialsEntry node is connected via a short path to nodes labeled meshes, which represent annotations with MeSH concepts.

From there, we enter the MeSH ontology. MeSH concepts are represented by MeshClass nodes, which are connected via subClassOf relationships. The graph model representing the UMLS is similar to the MeSH subgraph. It consists of UMLSconcept nodes connected by hierarchical child and parent relationships according to the structure of the data source.

Medical data models are represented according to their hierarchical structure. The entry point is a Study node. A medical data model’s hierarchical structure is represented by a path starting from the Study node and continuing to nodes labeled StudyEvent, Form, ItemGroup, and Item. Item nodes are connected to Alias nodes, which are then connected to their respective UMLSconcept nodes. A more detailed description of the MDM subgraph can be found in Multimedia Appendix 1.

#### Cross-Domain Linking of Study Data

**Figure 5.**
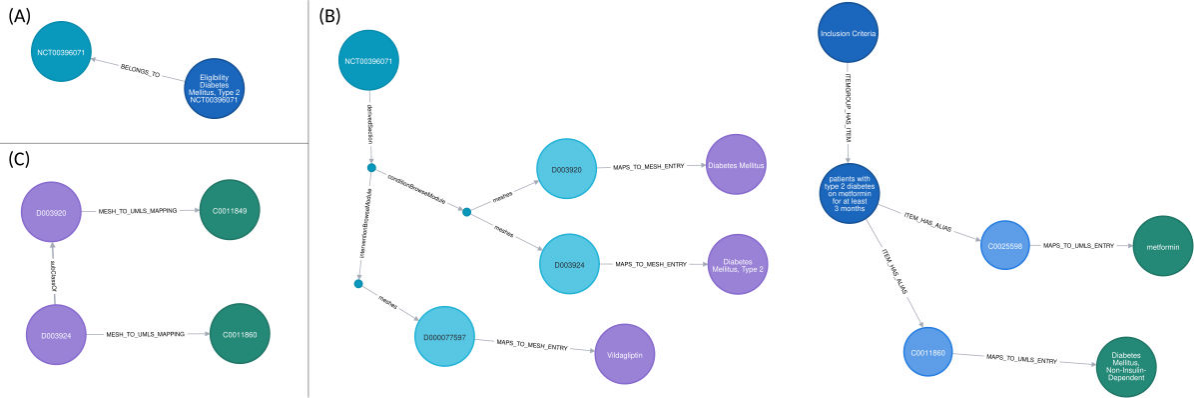
Linking of data in the graph. Nodes are color-coded according to their data source, with turquoise indicating nodes belonging to the ClinicalTrials.gov subgraph, dark blue indicating nodes belonging to the MDM Portal subgraph, purple indicating MeSH nodes, and green indicating Unified Medical Language System (UMLS) nodes. (A) ClinicalTrials.gov and MDM Portal. Complementary study forms from the MDM Portal are linked to the respective ClinicalTrials.gov study, which shares the same National Clinical Trial identifier. The nodes are connected via BELONGS_TO relationships. (B) Annotations and ontologies. Annotations of the study resources (ClinicalTrials.gov and MDM Portal) are linked to their corresponding representations in the respective ontologies (MeSH and UMLS) via MAPS_TO_MESH_ENTRY or MAPS_TO_UMLS_ENTRY, respectively. (C) Cross-mappings between ontologies. MeSH and UMLS codes representing the same biomedical concept are linked via a MESH_TO_UMLS_MAPPING relationship. Here, the codes D003920 and C0011849 both represent the same concept (“diabetes mellitus”). Similarly, D003924 and C0011860 also represent the same concept.

Our knowledge base integrates study-related data from heterogeneous sources and domains. It connects studies with related study forms, study annotations with biomedical ontologies, and links concepts across ontologies (Figure 5):

- ClinicalTrials.gov and the MDM Portal: Entries from ClinicalTrials.gov are linked to corresponding medical data models from the MDM Portal. These medical data models include structured versions of studies’ eligibility criteria, related data dictionaries, base datasets, and other study forms. In the graph, the main node of a study is linked to the main node of the related medical data model. This type of link allows users to explore additional information of a study, such as study data dictionaries or case report forms that are not available in ClinicalTrials.gov alone.
- Annotations and ontologies: Studies and medical data models in ClinicalTrials.gov and the MDM Portal are annotated with concepts from ontologies, such as MeSH and UMLS. In the graph, we link these annotations to their corresponding representations in the respective ontologies. For instance, the MeSH annotation nodes of the ClinicalTrials.gov study entries, which represent MeSH concepts, are connected to matching nodes in the MeSH ontology graph. Similarly, the annotation nodes of the MDM Portal graph are linked to matching UMLS nodes. This enables users to explore not only direct annotation matches in the ontology but also semantically related concepts, such as broader or narrower terms.
- Cross-mappings between ontologies: Links between concepts from the UMLS and MeSH represent the same biomedical concept appearing in both ontologies. This connects terms across ontologies and helps unify annotation references to equivalent concepts in different ontologies. More precisely, a CUI in the UMLS represents a biomedical concept by aggregating synonymous terms from other vocabularies, such as MeSH. It can map to multiple terms from one or more vocabularies. Therefore, the mapping between ontologies in our graph is not one-to-one but can link one UMLS concept to multiple MeSH concepts.

Semantic annotations of data in ClinicalTrials.gov and the MDM Portal with terms from biomedical ontologies, such as MeSH or the UMLS, enable a precise description of the data. However, the full potential of such annotations is realized through the incorporation of the ontologies’ hierarchical structure. By incorporating the complete structure of the ontology in the BRAinS-Graph, we expand the knowledge about existing semantic annotations and enable semantic structure queries. Together, the connections in the knowledge base enable cross-domain querying and semantic exploration of study data across different domains. Users can now trace relationships from a study to its related forms, its structured eligibility criteria, or to semantically related studies, in a single knowledge base. This facilitates more powerful and comprehensive cross-domain querying, enhancing study findability and exploration.

### Mapping Statistics

Here, we provide quantitative information on the coverage and quality of the mappings between data sources. In the example set of the diabetes studies in Germany as of February 2025, we were able to create links between the different data sources based on the previously described mappings.

In total, 7 distinct ClinicalTrials.gov studies are linked to at least one medical data model from the MDM Portal. In turn, 42 medical data models were linked back to studies. We recognize that these numbers are relatively small compared to the size of the dataset. However, the fact that we were able to link studies with medical data models shows the feasibility of our approach. The number could be improved by a more targeted integration of medical data models, taking the NCT identifiers of ClinicalTrials.gov studies as the search input for downloading data from the MDM Portal.

Better coverage was achieved with the ontology-based mappings. Of 117,093 Alias nodes, meaning UMLS annotations of medical data models, representing 12,799 distinct UMLS concepts, 69,495 (59%) could be linked to respective UMLS nodes. Some Alias nodes link to the same UMLS node, with a total of 6954 (54%) distinct UMLS concepts linked. This corresponds to a mapping coverage of 59% (69,495/117,093) of Alias nodes and 54% (6954/12,799) of distinct UMLS concepts used in the annotations.

The mapping of MeSH annotations from ClinicalTrials.gov studies to their respective concepts in the MeSH ontology concepts involved linking 4044 (99.8%) of 4052 MeSH annotations to their respective concepts. Of the 665 distinct MeSH concepts used in the annotations, 663 (99.7%) could be linked to ontology nodes, resolving 99.7% (663/665) of MeSH annotations to ontology nodes.

### Clinical Applications

#### Overview

The BRAinS-Graph is available as a novel tool for health study exploration. It is a source for the overall analysis of ongoing studies, including the distribution of study types, regional and socioeconomic differences across Germany, or the diseases targeted. The graph also offers novel ways to search for and explore health studies; for example, patients get the opportunity to find studies relevant to their own patient history, and clinicians can quickly scan the ongoing study landscape. In this section, we introduce use cases that highlight these querying capacities. All use cases refer to the previously described data set of diabetes studies in Germany as of February 2025. In Multimedia Appendix 1, we provide more detailed information contrasting the use cases of the BRAinS-Graph with what can and cannot be done in the native interfaces of the data sources.

#### Statistical Analyses

##### Study Type Distribution for Meta-Analysis

Meta-analyses require a comprehensive understanding of the type of studies to be incorporated into the analysis. Studies are commonly distinguished as interventional and observational studies. In ClinicalTrials.gov, interventional studies are classified by randomization (randomized vs nonrandomized) and further categorized by more detailed design, including parallel, cross-over, sequential, and single-group studies. Analogously, observational studies are divided into prospective, retrospective, and cross-sectional designs. These categories are further refined into cohort, case-control, and case-only studies. Here, we analyze the distribution of these study types in our knowledge base. It is important to note that we consider the classification of study types as provided in the source data and did not alter the classification for our analysis.

**Figure 6.**
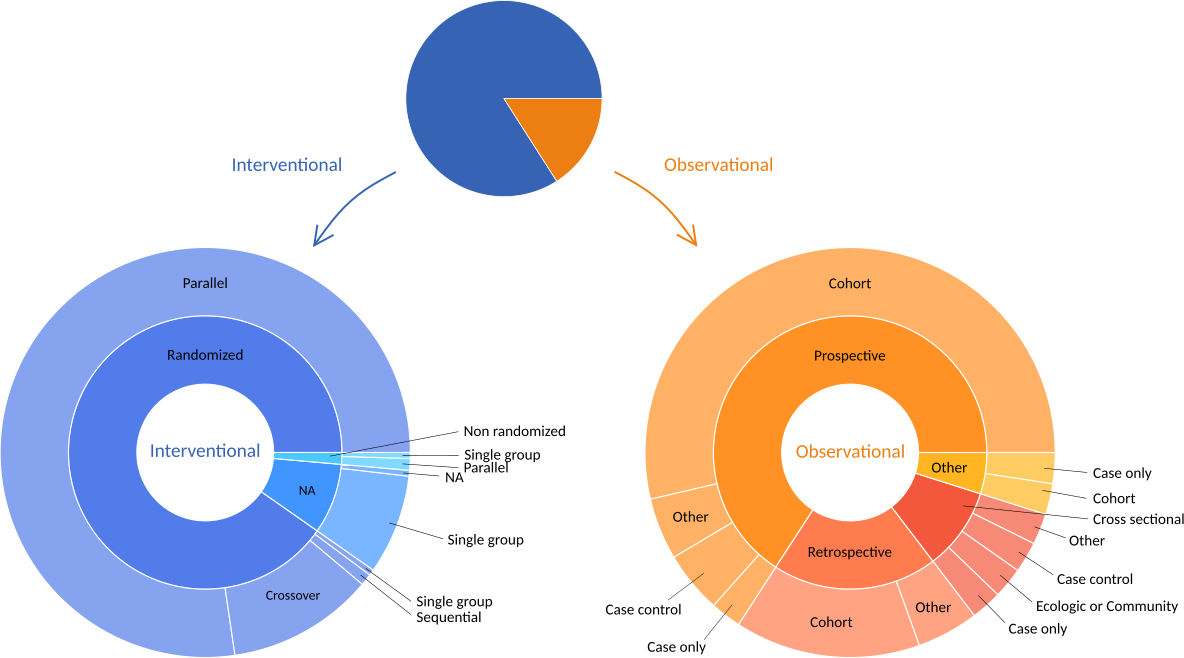
Sunburst charts of study type distribution. The chart shows the overall proportion of interventional and observational studies in the dataset. The left chart (blue) further differentiates interventional study designs, with labels indicating the study design categories from the center to the outer circle. The right chart (orange) analogously displays the distribution of observational study designs. NA: not applicable.

To analyze the distribution of study types for supporting meta-analyses, we queried the knowledge base by matching the relevant nodes of the ClinicalTrials.gov subgraph that contain the *studyType* property. Specifically, we counted the number of these nodes according to their respective *studyType* values. We then refined the query to retrieve more detailed information about the study designs by including additional properties, such as *observationModel*, *interventionModel*, *timePerspective*, and *allocation*. The Cypher queries used for this analysis are provided in Listing S4 in Multimedia Appendix 1. An overview of the study types represented in the knowledge base is shown in Figure 6. In total, the knowledge base contains information on 1140 interventional and 179 observational studies.

This structured presentation of study data may assist researchers in designing meta-analyses that include evidence from both interventional and observational studies. To conduct a meta-analysis, it is essential to obtain a comprehensive overview of the available studies, their corresponding study types, and the number of studies in each category. The representation of study design information supports this process by providing a detailed view of the types and frequency of both interventional and observational studies. This can help researchers assess the current state of research and make informed decisions about study inclusion and comparability.

##### Regional and Socioeconomic Distribution of Study Centers in Germany

To analyze the distribution of diabetes studies in Germany that are actively recruiting participants, we extracted the geolocations of 126 cities hosting study centers from the knowledge base. We then mapped the number of recruiting studies per city onto a geographic map of Germany to visualize regional variation. The number of recruiting studies per location ranges up to 31, with Berlin having the highest count of studies. In contrast, several cities have only one registered study.

**Figure 7.**
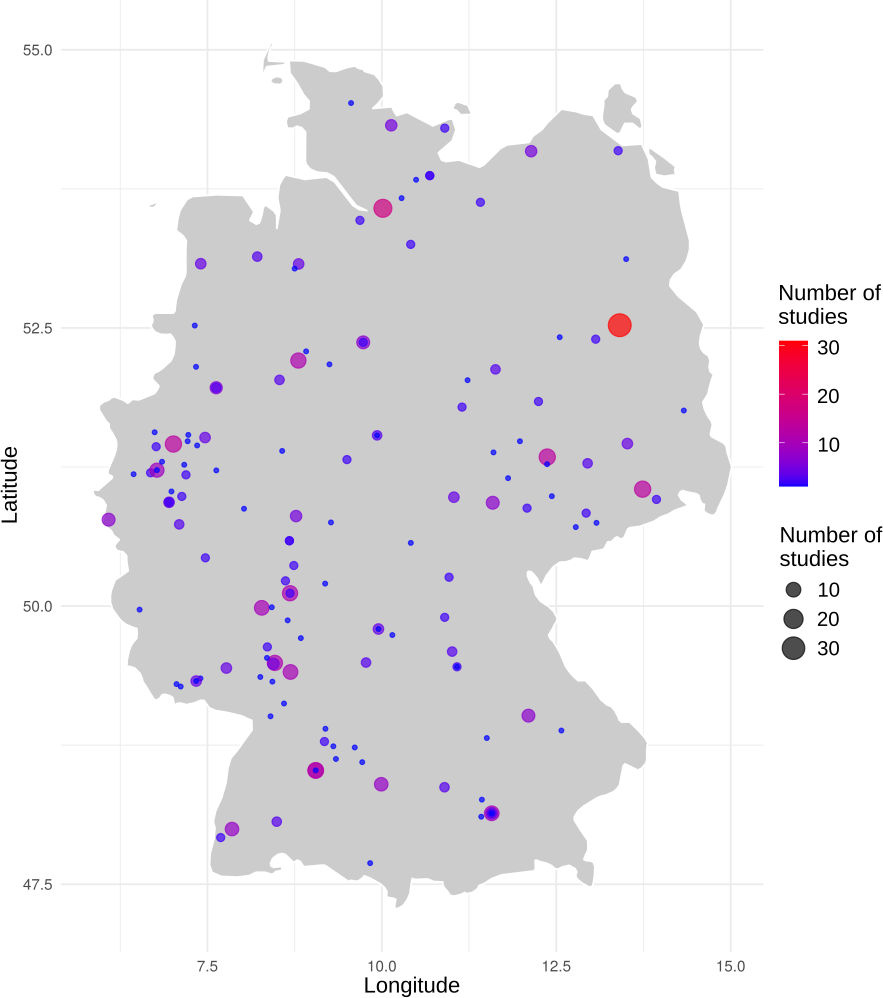
Geographic distribution of actively recruiting studies in Germany. The number of studies per city is shown on the map. Circle size and color intensity correspond to the number of studies per city. Coordinates are based on the study location information provided in ClinicalTrials.gov. The map highlights clusters in major urban areas and disparities between regions.

As shown in Figure 7, study center locations are primarily clustered in the west and southwest of Germany. Conversely, the region between Hamburg, Hanover, Greifswald, and Berlin in the north and northeast of Germany has very few actively recruiting diabetes studies. Notably, this regional distribution appears to reflect the geographic distribution of German university hospitals [29], suggesting a potential regional underrepresentation of research, particularly clinical studies, in the field of diabetes. For example, the region of Western Pomerania, which is sparsely populated without major cities, falls within the aforementioned underrepresented region and has only a limited number of study centers. While this may be expected based on population density, future analyses should consider the potential underrepresentation of people from this region when interpreting study results.

The proposed map may assist in identifying underrepresented regions and assessing the need for the initiation of new study centers. It also suggests that studies typically focus their recruitment efforts on densely populated areas and larger cities in particular. While this is understandable given the advantages in infrastructure, such as a larger pool of study participants with greater mobility and availability of facilities and equipment for data collection and patient examination, it may contribute to a gap in the heterogeneity of study populations. For example, disadvantages in the socioeconomic situation, referred to as regional socioeconomic deprivation, impact factors such as life expectancy, mortality, and health risks [30]. Specifically, rural areas in the north and northeast of Germany have high socioeconomic deprivation [31], including our previous example of Western Pomerania.

The “urban-centric” distribution of study centers throughout Germany may benefit people living in cities, who, one could argue, are therefore more likely to voluntarily participate in clinical studies, resulting in their overrepresentation in study data. This leads to an unequal representation of the population’s overall socioeconomic background in clinical studies and may therefore impact the generalizability of study findings. To rectify this potential bias, one should consider the distribution of study centers with respect to contextual indicators such as the *German Index of Socioeconomic Deprivation* [31].

Further analyses are needed to examine the representation of real-world regional population disparities in study populations and the resulting implications for the planning of future studies.

##### Revealing Disparities in the Distribution of Academia and Industry Studies

To analyze study sponsorship, we queried the knowledge base for information regarding industry and academia involvement in studies. We found that industry outweighs other players in sponsoring diabetes studies in Germany, especially in interventional studies. Approximately 65% (851/1319) of all studies in our knowledge base have an industry-lead sponsor. Studies with at least one industry collaborator account for approximately 16% (217/1319) of all studies, of which 139 (139/1319, 11%) also list an industry-lead sponsor. Due to limitations in the available metadata, we could not retrieve equivalent information about academia involvement because lead sponsors of the remaining 35% (468/1319) of all studies are classified under broader categories [32]. These include not only academic institutions but also various potential nonindustry sponsors. Further exploration of lead sponsor information, such as their names, may facilitate a more granular classification of sponsorship, but this is beyond the scope of this work. Nevertheless, the prevalence of industry-sponsored studies is apparent, with approximately two-thirds of all studies receiving sponsorship from industry. This suggests that the majority of studies are industry-driven, in contrast to those originating from academic institutions, such as university hospitals, and highlights a high level of industry engagement in the diabetes research landscape.

To further explore the role of industry sponsorship, we queried the knowledge base for the industry-specific study sponsors and their engagement in diabetes-related research studies conducted in Germany (see Listing S5 in Multimedia Appendix 1). Notably, companies well known for their diabetes research, such as *Novo Nordisk A/S* and *Eli Lilly and Company*, are among the most frequent sponsors, each supporting over 80 and 70 studies, respectively. Table 2 lists the top 5 industry sponsors by number of studies sponsored.

**Table 2.** Top industry sponsors^a^.

Sponsor name	Sponsored studies, n
Novo Nordisk A/S	81
Eli Lilly and Company	71
Boehringer Ingelheim	56
Sanofi	53
AstraZeneca	50

a The table shows the top 5 study sponsors from industry, ranked by the number of studies they sponsor. The counts reflect the number of sponsored studies included in the BRAinS-Graph used for the analysis.

### The Patient’s Perspective: Identifying Relevant Studies Through Eligibility Criteria

Patients, or physicians, looking for studies that could benefit the patient’s outcome typically look for studies that (1) investigate the patient’s specific disease, (2) are actively recruiting participants, and (3) have study centers located within a reasonable distance.

**Figure 8.**
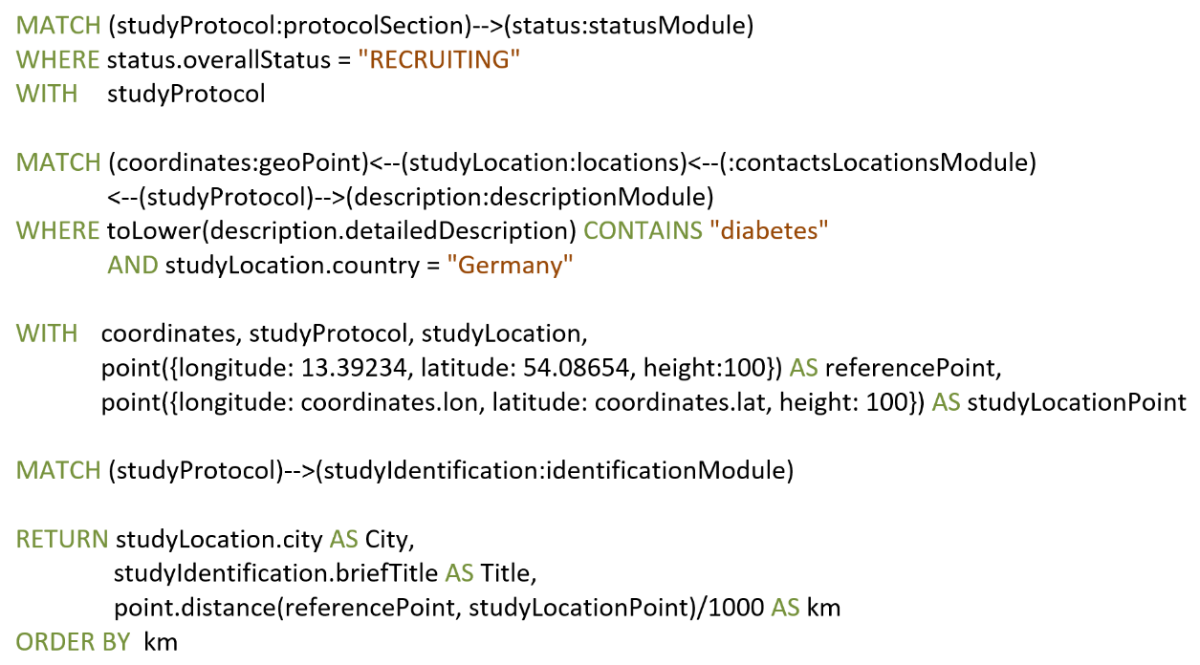
Querying for recruiting diabetes studies in Germany. The query first matches recruiting studies. It then matches locations and descriptions of these studies and filters for descriptions containing the word “diabetes” and locations within Germany. A reference point is defined with specific coordinates, while each study’s coordinates are saved as study location points. The final MATCH clause matches the studies’ identification module, which is then used to return each study’s brief title (Title), together with the study’s location (City), and the distance from the reference point (km), with results ordered by proximity (Listing is included in Multimedia Appendix 1).

**Figure 9.**
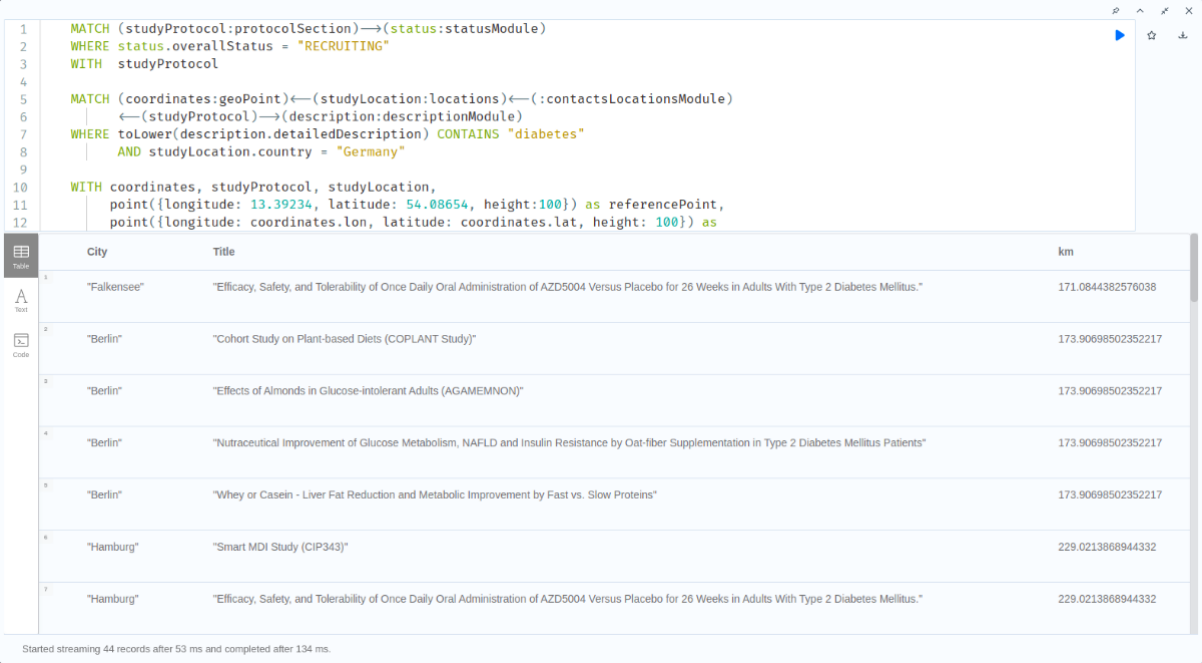
Neo4j browser view of the query from Figure 8 and its results. The output shows study locations and titles of recruiting diabetes studies in Germany, ranked by distance to a reference location.

Imagining a fictitious patient from the region of Greifswald (northeastern Germany), we search for that person’s treatment options for diabetes. Note that both “Greifswald” and “diabetes” can be replaced with any other location and disease of interest. The query in Figure 8 retrieves all studies that meet the specified criteria. Specifically, the study description must contain the word “diabetes,” and the study status must be “recruiting.” The query returns a ranked list of matching studies according to their distance to the given coordinates of Greifswald. A subset of the resulting output is shown in Figure 9, with study centers in Falkensee and Berlin as the closest options. The complete output is available in Table S1 in Multimedia Appendix 1.

Disease-specific querying and location-based filtering provide a first, comprehensive list of potentially suitable studies. In the next step, the patient’s eligibility for inclusion as a study participant needs to be assessed. Therefore, we leverage the integrated structured eligibility criteria originating from the MDM Portal for our graph-based search. This extended search query incorporates the patient’s eligibility based on the study’s inclusion and exclusion criteria. We mapped structured eligibility criteria from the MDM Portal to the respective studies from ClinicalTrials.gov in the BRAinS-Graph, as described in the BRAinS-Graph knowledge base section, “Cross-domain linking of study data.” We based our mapping on the NCT identifier from ClinicalTrials.gov. The direct connection between these data items in the graph enables sophisticated querying of the studies’ eligibility criteria, provided that the respective study is included in the BRAinS-Graph.

**Figure 10.**
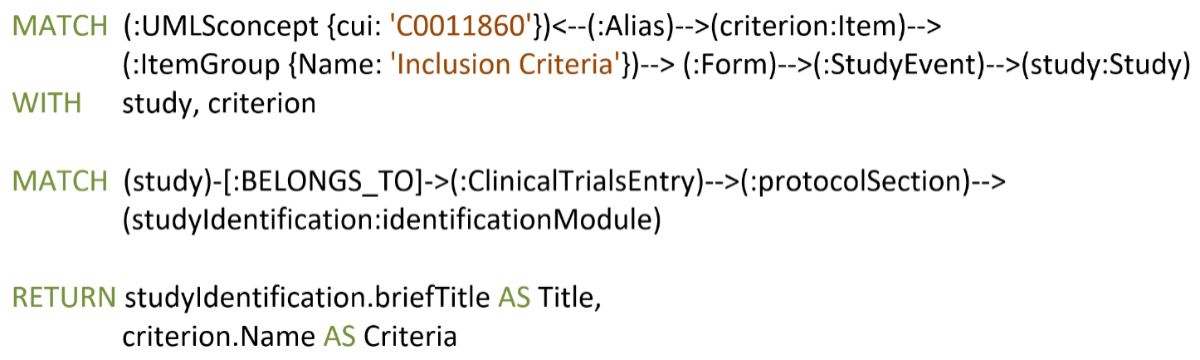
Querying for eligibility criteria. The query matches the Unified Medical Language System (UMLS) concept with the concept unique identifier (CUI) C0011860 (“diabetes mellitus”). The first MATCH clause matches the path from this UMLS concept to a study, specifying that the ItemGroup represents an inclusion criterion. The ItemGroup corresponds to a group of items or criteria. The matched study is used in the second MATCH clause to query for the study’s identificationModule. The query finishes by returning the brief title of the study (Title) and the inclusion criteria (Criteria) per study title (Listing is included in Multimedia Appendix 1).

The integration of structured inclusion and exclusion criteria together with their semantic annotations and the UMLS hierarchy offers new opportunities for searching specifically for a disease in the inclusion criteria. Considering the example of diabetes, we start with the UMLS term for “Diabetes Mellitus” and specify that we are searching for studies that list the prevalence of diabetes mellitus as an inclusion criterion for participation in the study. In our graph-based approach, this means querying for the UMLS node C0011860 (“Diabetes Mellitus”) and inclusion criterion nodes connected to this specific UMLS node. Conceptually, this represents the semantic annotation of the criteria with the respective UMLS term. To ensure comparability, we apply the criteria from the previous query and then incorporate the UMLS code for diabetes mellitus instead of looking for the word “diabetes” in the study’s description. We further declare the disease to be an inclusion criterion. Translated to our graph, this means that the ItemGroup nodes representing the inclusion criteria should be connected to the respective UMLS node. The respective query is shown in Figure 10.

**Figure 11.**
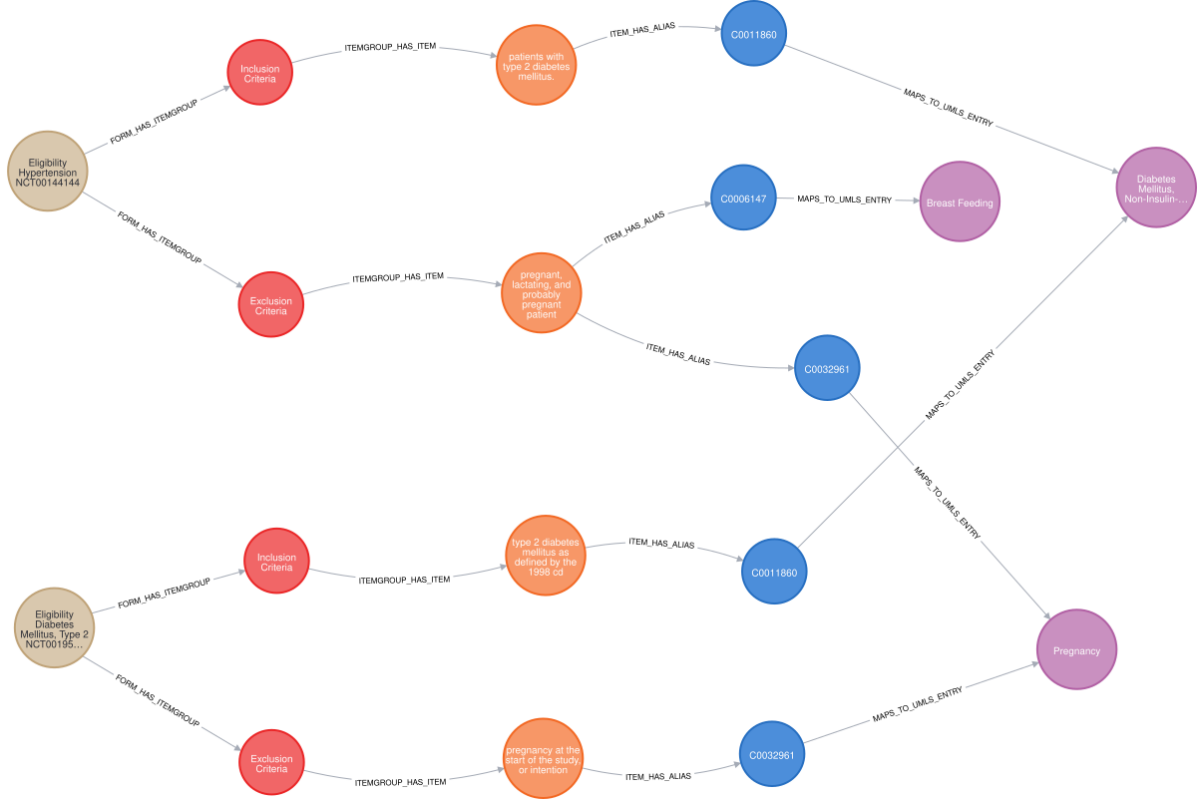
Graph representation of 2 diabetes studies excluding pregnant patients. Nodes representing eligibility criteria (beige) connect via nodes specifying inclusion or exclusion criteria (red) to nodes representing individual criteria (orange), which are annotated (blue) and linked to the corresponding Unified Medical Language System (UMLS) concepts (purple) for diabetes and pregnancy. The shared UMLS concepts highlight common eligibility criteria between the 2 studies.

We now extend the query to account for exclusion criteria, for example, to find studies studying diabetes that exclude pregnant patients. Figure 11 illustrates the graph representation of 2 studies that fulfill these criteria. Note that the UMLS nodes (purple) representing diabetes and pregnancy are connected to the study nodes (beige) via a series of edges, or relationships. These relationships connect the UMLS nodes to the studies’ inclusion or exclusion criteria, that is, the *Item* nodes (orange). The *ItemGroup* nodes (red) between the study nodes and the *Item* nodes specify whether it is an inclusion or exclusion criterion. The graph illustrates that the 2 studies share common eligibility criteria, indicating a degree of similarity between the studies in an intuitive visualization.

### Technical Applications

#### Overview

The knowledge base facilitates various applications. While the preceding section focused on clinical applications, this section highlights technical use cases of the knowledge base. Again, all analyses are based on the dataset of diabetes studies in Germany as of February 2025, and the use cases are contrasted with the capabilities of the native interfaces of the data sources in Multimedia Appendix 1.

#### Analyzing Study Information

**Figure 12.**
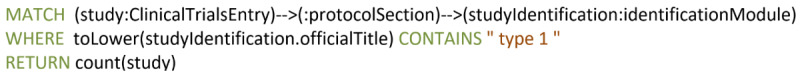
Querying for type 1 diabetes studies. The query matches studies and their identification module, then filters for studies whose official title contains the words “type 1 diabetes” and returns the count of matching studies (Listing is included in Multimedia Appendix 1).

The objective of this example is to demonstrate how the knowledge base can be used to analyze the annotation accuracy of studies from ClinicalTrials.gov. The example focuses on type 1 diabetes. To this end, we selected the set of studies whose official titles contained the name of the disease or a variation of the name. Manual review of the query results was used to iteratively refine the query. The query refinement included adding a space in front of “type” to exclude false positives, such as NCT01591460, whose official title contains “Genotype 1.” Additionally, we defined the query to include “type 1” without adding the word “diabetes” to cover naming variants such as “type 1 DM.” This refinement resulted in a set of 116 studies, as returned by the query in Figure 12.

**Figure 13.**
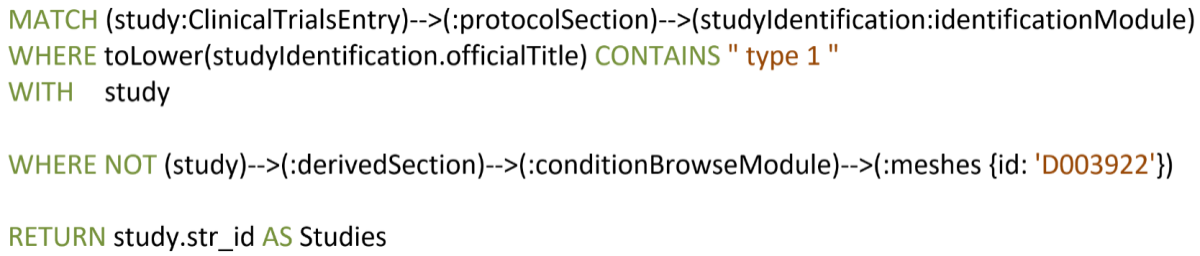
Type 1 diabetes studies without the corresponding MeSH annotation. The query matches studies and their identification module, then filters for studies whose official title contains the words “type 1 diabetes.” It then excludes studies that have a connecting path to the MeSH term D003922, representing an annotation with that MeSH term. The query returns the study identifier (Studies) for all matching studies (Listing is included in Multimedia Appendix 1).

We then queried this set for their MeSH annotations. The set of terms used for annotation consists of 13 distinct MeSH terms. In our analysis, we focus on the following 3 MeSH terms: D003920 (“Diabetes Mellitus”), D003922 (“Diabetes Mellitus, Type 1”), and D003924 (“Diabetes Mellitus, Type 2”). Among the 116 studies, 108 (93%) are annotated with the MeSH term D003922, meaning that 8 (7%) studies are not annotated with the respective MeSH term for type 1 diabetes, although the disease is mentioned in their title. The query in Figure 13 returns these studies, which could be used for further investigation into why the MeSH term is missing. They could also be used as suggestions for adding the respective MeSH term for improving annotation.

**Figure 14.**
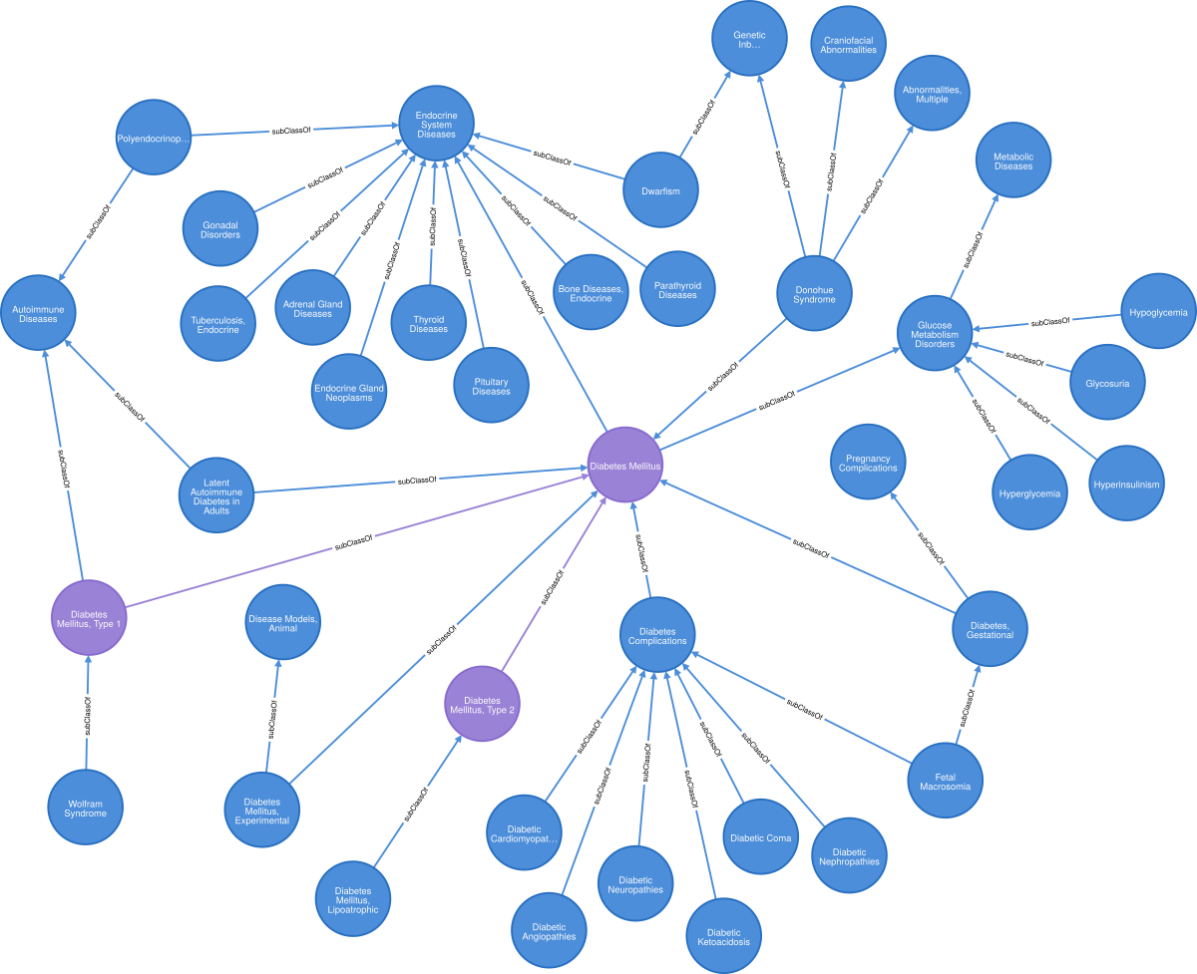
Excerpt of the graph representation of MeSH. The parent-child relationships of the MeSH terms for diabetes mellitus and its child terms type 1 and type 2 diabetes mellitus are highlighted in purple. Nodes are connected via subClassOf relationships, visualizing the hierarchical structure of MeSH.

**Figure 15.**
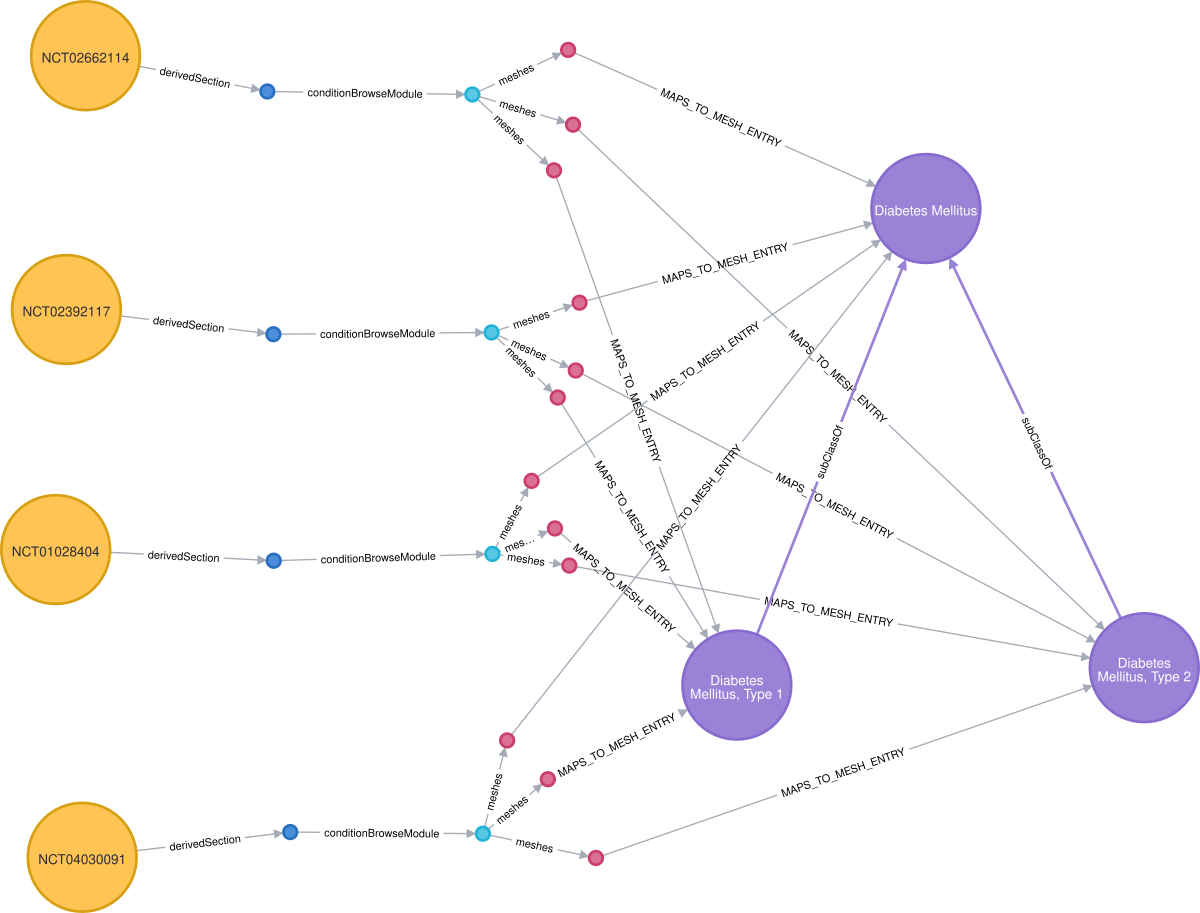
Studies annotated with MeSH terms. Four studies (yellow) are linked to 3 MeSH terms (purple) representing diabetes mellitus, type 1 diabetes, and type 2 diabetes. The graph shows the relationships between studies and their corresponding MeSH annotations.

We examined the annotations of these 8 studies in more detail. A question of particular interest was whether these studies are at least annotated with the broader MeSH term “Diabetes Mellitus.” As type 1 diabetes is a specification of diabetes mellitus, these 2 terms are linked by a parent-child relationship in the MeSH hierarchy. The BRAinS-Graph facilitates the visualization of this hierarchical relationship (Figure 14). Consequently, we checked the 8 studies for annotation with the broader disease term. We found that 1 (12.5%) of the 8 studies had no MeSH annotations at all. Two (25%) studies are annotated but not with the term “Diabetes Mellitus.” In total, only 5 (62.5%) of the 8 studies were annotated with “Diabetes Mellitus.” An overview of the 8 studies and their respective annotations is provided in Table 3. It is evident that these studies are generally sparsely annotated, with zero to a maximum of 2 MeSH terms. Similarly, we checked for annotations with “Diabetes Mellitus, Type 2.” Four (3%) studies are annotated with this term. Interestingly, visualization of the studies’ annotations in the knowledge base shows that these 4 studies are also annotated with both the MeSH terms for diabetes mellitus and type 1 diabetes (Figure 15).

**Table 3.** MeSH term annotations for 8 selected studies^a^.

Study identifier	MeSH terms
NCT00139659	Diabetes Mellitus, Asthma
NCT01060917	Diabetes Mellitus
NCT01120444	Diabetes Mellitus
NCT02155023	—^b^
NCT02585778	Hypercholesterolemia
NCT03175315	Diabetes Mellitus
NCT03781232	Diabetes Mellitus
NCT06025513	Insulin Resistance

a These studies are not annotated with the MeSH term for type 1 diabetes. The table lists each study identifier and its associated MeSH terms, showing that annotations are sparse and that not all relevant studies are tagged with the broader term “Diabetes Mellitus.”

b Not applicable.

## Discussion

### Principal Results

Finding relevant studies is a prerequisite for many clinical applications, yet it is often hindered by the distribution of partially unstructured study information across resources and data domains. As findability is closely linked to the effective search, exploration, and retrieval of health studies, this distribution presents a substantial challenge. Our work addresses this issue. The BRAinS-Graph framework is designed to integrate different study resources into a Neo4j graph database. Consisting of 4 modules, it integrates ontologies (MeSH and UMLS) and study metadata from ClinicalTrials.gov and the MDM Portal.

The framework is developed for integrating structured, machine-readable, and annotated study forms, especially eligibility criteria, with broader study metadata. By linking MDM Portal data, ClinicalTrials.gov records are enriched with ontological knowledge from the UMLS annotations provided by the MDM Portal. This enables users to search for studies using UMLS terms, facilitating the exploration of supplementary study information not currently available in ClinicalTrials.gov. The resulting knowledge graph, referred to as the BRAinS-Graph, provides a basis for study analysis and exploration for future users, including clinicians, researchers, and patients. The use cases presented here demonstrate the spectrum of clinical and technical applications accessible through the BRAinS-Graph, offering readers a concrete understanding of its capabilities. While these examples are informative, the current implementation requires some familiarity with Python programming and code repositories for setup, and basic knowledge of graph databases is beneficial for querying the BRAinS-Graph. Nonetheless, we have kept technical requirements as accessible as possible by providing detailed documentation in the public code repository. Once deployed, the graph functions as a foundation for implementing further analysis and retrieval methods. We strongly believe that providing a framework to build such a database is an important and necessary advancement.

We remind the reader that the results presented here are just an example. Further diseases can be analyzed following the documentation available in the public repository. Owing to its free, open-source, and modular architecture, the framework is reusable and extendable, making it adaptable for the development of individual knowledge bases. Although our use cases focus on diabetes, it is important to note that the framework is generic and disease independent. It can be used to create integrated study graphs for any set of studies, regardless of the disease, geographic region, or other research interests being studied.

Our work contributes to improved study findability by providing a framework that supports the search for, exploration of, and retrieval of health studies. Improving study findability, and consequently enhancing the reusability of study data, is a step toward achieving the FAIR principles in clinical research. The main aspects addressed in this work are findability and reusability. Other FAIR principles, that is, accessibility and interoperability, are touched upon but not addressed in detail. The integration of ontological knowledge through terminology enrichment can be seen as a contributing factor to these aspects. However, more work remains necessary.

### Limitations

Despite these contributions, limitations remain. Our work relies on the quality of the data provided by the external resources. ClinicalTrials.gov experiences several challenges regarding the data quality of their study entries [33], many of which still need to be addressed. Ensuring the quality of the source data remains a first imperative step toward meaningful study analysis.

While the querying capabilities of the BRAinS-Graph are powerful, results must be validated by domain experts before the graph can be implemented in real-world settings. Although we have constructed a highly accessible graph, quantifying its contribution to the FAIRness of studies remains future work. To properly evaluate its impact, a comprehensive FAIR assessment [34] should be conducted, following examples such as the evaluation of the NFDI4Health (National Research Data Infrastructure for Personal Health Data) Health Study Hub [35,36].

This work serves as a prototype for graph-based study discovery, illustrating a technical vision for broader implementations of comprehensive study graphs. While the BRAinS-Graph is built on a technically feasible approach that links studies across domains, it currently relies on limited primary study data sources. In practice, not all relevant studies conducted at German university hospitals are necessarily available in these data sources, and this may remain the case in the future. However, the BRAinS-Graph could be implemented locally within individual clinics and connected to their internal study databases in the future. Clinics could then host their own customized study graphs linked to their internal databases, thereby providing an informative tool for their clinical researchers and physicians to search, explore, and compare relevant studies more effectively.

### Outlook

Moreover, our approach is a first step toward a study recommendation system. External study data from resources, such as national registries, could be leveraged to recommend relevant or similar studies within the local study graphs. On a larger scale, the same graph-based approach could be adopted by national initiatives such as the Network University Medicine [32], facilitating cross-institutional linkage and enhanced study discovery across Germany by reusing the BRAinS-Graph framework in the Network University Medicine study network. We have already demonstrated the feasibility of this vision by developing a graph-based representation of the NFDI4Health Health Study Hub [35], as described in a study by Gütebier et al [37].

Several directions for further research and development remain, including evaluating the framework’s scalability, its application in targeted clinical contexts, and the development of advanced similarity search functionalities. Our work establishes a foundation for a graph-based similarity search capable of not only identifying studies of interest but also of detecting studies with comparable features [38]. These capabilities have the potential to guide the development of new studies, support the reuse of study designs, facilitate the matching of patients to study populations, and, ultimately, expedite participant recruitment. Although these visions have yet to be realized, the BRAinS-Graph represents a novel and meaningful contribution to enhancing the discovery, accessibility, and practical utility of health studies.

### Conclusions

We developed the BRAinS-Graph, a semantically enriched knowledge base that integrates data from ClinicalTrials.gov, the MDM Portal, MeSH, and UMLS into a unified graph database. By linking study entries with complementary medical data models and ontological information, it supports cross-domain searches of study metadata, eligibility criteria, and structural information. The framework enhances the findability and reusability of health studies, providing a reusable foundation for future applications, such as similarity searches and study recommendation systems.

## Supplementary material

10.2196/86812 Multimedia Appendix 1 Supplementary materials including graph design, an overview of related study resources, additional listings of Cypher queries, an additional use case, a contrast of key scenarios with the native interfaces of ClinicalTrials.gov and the MDM Portal, a how-to guide, an overview of technical requirements, and a validation on a new data set.

## Abbreviations

BRAinS-Graph: Biomedical Knowledge Graph for Recommending and Analysing Health Studies
CDISC ODM: Clinical Data Interchange Standards Consortium Operational Data Model
CUI: concept unique identifier
ETL: extract-transform-load
FAIR: findability, accessibility, interoperability, and reusability
FLOSS: Free, Libre, and Open Source Software
MDM Portal: Portal for Medical Data Models
NCT identifier: National Clinical Trial identifier
NFDI4Health: National Research Data Infrastructure for Personal Health Data
NUM: Network University Medicine
OID: Object Identifier
UMLS: Unified Medical Language System
WHO: World Health Organization
