# Supplementary material for: Enhanced Health Study Discoverability: Graph-Based Analysis Approach
Source: JMIR Med Inform. 2026 Aug 18;14:e86812. doi: 10.2196/86812 (PMC13484817; doi:10.2196/86812)
Supplement: Supplementary file 1 [file medinform-v14-e86812-s001.docx]

Supplementary Materials for “A graph-based analysis approach for enhanced health study discoverability”

# Graph design

## Graph schema visualizations

The graph schema of the BRAinS-Graph for the data sources ClinicalTrials.gov and the MDM Portal is shown in detail in Figures S1 and S2, respectively.

Figure S1 Detailed schema of the ClinicalTrials.gov subgraph. Node properties are omitted for readability purposes. Nodes are grouped with respect to their labels, each group is represented by a color (eg, light green for nodes that store information about outcomes). The entry node is gray, labeled ClinicalTrials Entry. The schema shows the graph structure of a ClinicalTrials.gov study entry.

Figure S2 Detailed schema of the MDM Portal subgraph. Nodes are depicted as circles with node labels written inside the circles. Node properties are grouped next to each node. Directed relationships are shown as directed edges between circles, their relationship type written above each edge. The entry node has the label Study, coherent with the ODM definition. The schema shows the graph structure of an entry from the MDM Portal.

## Example schema description of the MDM Portal subgraph

The graph schema for medical data models from the MDM Portal has eleven distinct labels, as shown in Figure S2. Entry node for each form is a node with the label *Study*. The hierarchical structure of the forms is represented by the path of nodes with labels *Study*, *StudyEvent*, *Form*, *ItemGroup*, and *Item*. A study can have multiple forms, with each form consisting of multiple item groups, which themselves can have multiple items. Items are, for example, questions in a medical form, designed to collect a patient’s health parameters, such as lab values. Therefore, nodes with the label *Item* are connected to *MeasurementUnit*, *RangeCheck* and *CodeList* nodes, each defining the respective metrics for the form’s item. *CodeList* nodes are further connected to *CodeListItem* nodes, specifying values in the code list. *BasicDefinitions* nodes, representing base definitions in the ODM specification, are connected to *MeasurementUnit* nodes that represent measurement units defined in the base definitions. Connections to *Alias* nodes represent annotations with ontological terms.

# Related study resources

The German Central Study Hub of NFDI4Health is a collection of study information, initially developed for COVID-19 studies, aiming to make studies findable and accessible, while working towards FAIRification of the included studies [1]. The Study Hub combines data entries from ClinicalTrials.gov, the International Clinical Trials Registry Platform (ICTRP), the German Clinical Trials Register (DRKS), and manually collected study records. ClinicalTrials.gov is an established online registry of clinical research studies, including interventional and observational studies, hosted by the National Library of Medicine [2]. Studies are organized into study records associated with a set of data elements. The ICTRP^[1]^, initiated by the WHO, provides a “single point of access” search portal that collects multiple study registries. These registries adhere to the WHO Trial Registration Data Set^[2]^, a set of data elements describing the design, conduct, and administration of studies that need to be published in order for a study to be considered registered. The DRKS^[3]^ is a repository specific to Germany, with a collection of human clinical studies conducted in Germany [3].

Other notable study resources include the EU Clinical Trials Register^[4]^ and its successor the Clinical Trials Information System^[5]^, both on a European level, as well as the Clinical Research Metadata Repository^[6]^, and the ISRCTN registry^[7]^, specific to studies conducted in the UK.

In addition to these globally recognized resources, a multitude of smaller, local resources are managed, including the DZHK research platform [4]. The DZHK research platform is hosted by the German Center for Cardiovascular Research (DZHK) and presents a standardized and accessible data collection of studies conducted at or associated with the DZHK. Standardization is ensured by a Common Data Model, which includes a basic data set and a set of optional modules [4].

In addition to study registries, the Portal for Medical Data Models (MDM Portal) contains structural metadata, called medical data models [5,6]. The MDM Portal has been established as an important system in medical research by providing a significant metadata repository of medical forms, structured eligibility criteria, and study base data sets, summarized as medical data models. The portal manages and utilizes more than 25000 medical data models, which are consistently stored in the CDISC ODM format^[8]^ and annotated with UMLS terms (Unified Medical Language System) [7], ensuring standardization and semantic encoding [8].

# Listings from the main manuscript

The following listings are included in the main manuscript as figures.

Listing 1 Post-processing step of mapping MeSH terms to UMLS concepts. The query first matches all MeSH nodes and collects their distinct CUI identifiers into a list, which is then converted into a set to remove duplicates. It then matches UMLS nodes whose CUI appears in this set. Finally, the query creates MESH TO UMLS MAPPING relationships from each MeSH node to the corresponding UMLS concepts. The MERGE clause ensures that relationships are created only if they do not already exist, preventing duplicates.

MATCH (mesh:MeshClass)

WITH collect(distinct mesh.cui) AS mesh_cui_lists

WITH apoc.coll.toSet(apoc.coll.flatten(mesh_cui_lists)) AS mesh_cuis

MATCH (umls:UMLSconcept)

WHERE umls.cui IN mesh_cuis

MERGE (mesh)-[:MESH_TO_UMLS_MAPPING]->(umls)

MATCH (studyProtocol:protocolSection)-->(status:statusModule)

WHERE status.overallStatus = "RECRUITING"

WITH	studyProtocol

MATCH (coordinates:geoPoint)<--(studyLocation:locations)<--(:contactsLocationsModule)

<--(studyProtocol)-->(description:descriptionModule)

WHERE toLower(description.detailedDescription) CONTAINS "diabetes"

AND studyLocation.country = "Germany"

WITH	coordinates, studyProtocol, studyLocation,

point({longitude: 13.39234, latitude: 54.08654, height:100}) AS referencePoint,

point({longitude: coordinates.lon, latitude: coordinates.lat, height: 100}) AS studyLocationPoint

MATCH (studyProtocol)-->(studyIdentification:identificationModule)

RETURN studyLocation.city AS City,

studyIdentification.briefTitle AS Title,

point.distance(referencePoint, studyLocationPoint)/1000 AS km

ORDER BY km

Listing 2 Querying for recruiting diabetes studies in Germany. The query first matches recruiting studies. It then matches locations and descriptions of these studies and filters for descriptions containing the word “diabetes” and locations within Germany. A reference point is defined with specific coordinates, while each study’s coordinates are saved as study location points. The final MATCH clause matches the studies’ identification module, which is then used to return each study’s brief title (Title), together with the study’s location (City), and the distance from the reference point (km), with results ordered by proximity.

Listing 4 Querying for type 1 diabetes studies. The query matches studies and their identification module, then filters for studies whose official title contains the words “type 1 diabetes” and returns the count of matching studies.

MATCH (study:ClinicalTrialsEntry)-->(:protocolSection)

-->(studyIdentification:identificationModule)

WHERE toLower(studyIdentification.officialTitle) CONTAINS " type 1 "

RETURN count(study)

Listing 3 Querying for eligibility criteria. The query matches the UMLS concept with the CUI C0011860 (“Diabetes Mellitus”). A CUI is the UMLS Concept Unique Identifier. The first MATCH clause matches the path from this UMLS concept to a study, specifying that the ItemGroup represents an inclusion criterion. The ItemGroup corresponds to a group of items, or criteria. The matched study is used in the second MATCH clause to query for the study's identificationModule. The query finishes by returning the brief title of the study (Title) and the inclusion criteria (Criteria) per study title.

MATCH (:UMLSconcept {cui: 'C0011860'})<--(:Alias)-->(criterion:Item)-->

(:ItemGroup {Name: 'Inclusion Criteria'})--> (:Form)-->(:StudyEvent)-->(study:Study)

WITH	study, criterion

MATCH (study)-[:BELONGS_TO]->(:ClinicalTrialsEntry)-->(:protocolSection)-->

(studyIdentification:identificationModule)

RETURN studyIdentification.briefTitle AS Title,

criterion.Name AS Criteria

MATCH (study:ClinicalTrialsEntry)-->(:protocolSection)

-->(studyIdentification:identificationModule)

WHERE toLower(studyIdentification.officialTitle) CONTAINS " type 1 "

WITH	study

WHERE NOT (study)-->(:derivedSection)-->(:conditionBrowseModule)-->(:meshes {id: 'D003922'})

RETURN study.str_id AS Studies

Listing 5 Type 1 diabetes studies without the corresponding MeSH annotation. The query matches studies and their identification module, then filters for studies whose official title contains the words “type 1 diabetes”. It then excludes studies that have a connecting path to the MeSH term D003922, representing an annotation with that MeSH term. The query returns the study identifier (Studies) for all matching studies.

# Technical applications: Use of similarity measures

In order to analyze which data items are most commonly used in studies, we queried the UMLS terms and annotations. Therefore, we checked which key UMLS terms are shared by most studies. The three most commonly used UMLS terms in the annotation of study data from the MDM Portal are “Date in time” (*C0011008* used 1585 times), “Patients” (*C0030705* used 1022 times), and “Yes or No Response” (*C1512698* used 828 times). Query S1 counts the number of relationships going from all UMLS terms to other nodes. For a more detailed analysis, we investigate the most frequently used UMLS terms for the annotation of eligibility criteria. The most commonly used UMLS term for annotation of inclusion criteria are “Age” (*C0001779* used 332 times), “Informed Consent” (*C0021430* used 242 times), and “Gender” (*C0079399* used 104 times). While these terms mostly describe descriptive metadata, the UMLS terms used in the annotation of exclusion criteria are more disease-focused, as seen in the three most commonly used terms “Hypersensitivity” (*C0020517*), “Pregnancy” (*C0032961*), and “Comorbidity” (*C0009488*), each used 217, 205, and 171 times, respectively. The respective queries are shown in Listing S2.

```
MATCH (umls:UMLSconcept)<-[relation]-()
RETURN umls.cui AS CUI, umls.name AS Concept,
       count(relation) AS numberOfAnnotations
ORDER BY numberOfAnnotations DESC
```

Listing S1 Querying for the most commonly used UMLS terms in study annotation. The query matches all UMLS concepts and the connected relationships, each connected relationship representing a study annotation. For each concept, it returns the concept unique identifier (CUI), the concept name (Concept), and the total number of connected relationships (numberOfAnnotations), ordered by the number of connected relationships.

```
// Query for inclusion criteria
 MATCH (umls:UMLSconcept)<-[relation]-(:Alias)-->(:Item)
       -->(:ItemGroup {Name:'Inclusion Criteria'})
 RETURN umls.cui AS CUI, umls.name AS Concept,
        count(relation) AS numberOfAnnotations
 ORDER BY numberOfAnnotations DESC

// Query for exclusion criteria
 MATCH (umls:UMLSconcept)<-[relation]-(:Alias)-->(:Item)
       -->(:ItemGroup {Name:'Exclusion Criteria'})
 RETURN umls.cui AS CUI, umls.name AS Concept,
        count(relation) AS numberOfAnnotations
 ORDER BY numberOfAnnotations DESC
```

Listing S2 Most commonly used UMLS term for annotation of inclusion criteria and exclusion criteria. The first query matches UMLS concepts that are connected to Alias nodes, representing study annotations. The query only matches Alias nodes connected to Item nodes which in turn are connected to ItemGroup nodes representing inclusion criteria. For each concept, it returns the concept unique identifier (CUI), concept name (Concept), and the number of connected relationships (numberOfAnnotations), ordered by number of connected relations. The second query is analogous but matches ItemGroups representing exclusion criteria, returning the same set of properties.

We extend this high-level analysis of simple count queries with more deepened algorithmic queries. The underlying graph structure of the knowledge base motivates the use of graph algorithms for data analysis. Here, we analyze the annotation overlap between two example studies by calculating the Jaccard similarity of the studies’ eligibility criteria annotations. This simple measure is a first step in showing how similar the two studies are based on their eligibility criteria.

The example studies are the two studies from Figure 6 (*Graph representation of two diabetes studies excluding pregnant patients*) of the patient’s perspective use case in Section *The patient’s perspective: Identifying relevant studies through eligibility criteria*. We split the calculation of the Jaccard similarity for inclusion criteria and for exclusion criteria. The query used for calculating the score for the inclusion criteria is shown in Query S3. The Jaccard similarity score calculated for the inclusion criteria is 0.4, meaning out of ten UMLS terms used in the annotation of at least one of the two studies’ inclusion criteria, four UMLS terms are used in both studies. Computing the same similarity score for the studies’ exclusion criteria returns a significantly lower score of 0.06.

```
MATCH (study1:ItemGroup {Name: 'Inclusion Criteria'})<--(:Item)
       <--(:Alias)-->(umls:UMLSconcept)
 WHERE study1.StudyOID = "NCT00144144"
 WITH  study1, collect(id(umls)) AS study1Annotations

 MATCH (study2:ItemGroup {Name: 'Inclusion Criteria'})<--(:Item)
       <--(:Alias)-->(umls:UMLSconcept)
 WHERE study2.StudyOID = "NCT00195884"
 WITH  study1, study1Annotations,
       study2, collect(id(umls)) AS study2Annotations

 RETURN gds.similarity.jaccard(study1Annotations, study2Annotations) AS score
```

Listing S3 Compute Jaccard similarity for inclusion criteria of two studies. The query first matches the ItemGroup nodes representing inclusion criteria for the first study (with identifier NCT00144144), collects the connected UMLS concepts as annotations, and stores them as study1Annotations. It then repeats the same for the second study (with identifier NCT00195884), collecting UMLS concepts as study2Annotations. Finally, the query computes and returns the Jaccard similarity score between the two sets of annotations using the gds.similarity.jaccard function.

# Additions to main section *Clinical applications*

## Study type distribution for meta-analysis

The Cypher queries used in the analysis of the distribution of study types are provided in Listing S4.

```
// distribution of study types, interventional vs. observational
    MATCH (:ClinicalTrialsEntry)-->(:protocolSection)-->(studyDesign:designModule)
    RETURN studyDesign.studyType as StudyType,
           count(studyDesign.studyType) as NoOfStudies

// distribution of interventional studies, randomised vs. non-randomised
    MATCH (:ClinicalTrialsEntry)-->(:protocolSection)-->(studyDesign:designModule)
          -->(designInformation:designInfo)
    WHERE studyDesign.studyType = 'INTERVENTIONAL'
    RETURN studyDesign.studyType as StudyType,
           designInformation.allocation as Allocation,
           count(studyDesign.studyType) as NoOfStudies

// distribution of interventional studies further divided according to intervention model
    MATCH (:ClinicalTrialsEntry)-->(:protocolSection)-->(studyDesign:designModule)
          -->(designInformation:designInfo)
    WHERE studyDesign.studyType = 'INTERVENTIONAL'
    RETURN studyDesign.studyType as StudyType,
           designInformation.allocation as Allocation,
           designInformation.interventionModel as InterventionModel,
           count(studyDesign.studyType) as NoOfStudies

// distribution of observational studies, prospective vs. retrospective vs. cross-sectional
    MATCH (:ClinicalTrialsEntry)-->(:protocolSection)-->(studyDesign:designModule)
          -->(designInformation:designInfo)
    WHERE studyDesign.studyType = 'OBSERVATIONAL'
    RETURN studyDesign.studyType as StudyType,
           designInformation.timePerspective as Perspective,
           count(studyDesign.studyType) as NoOfStudies

// distribution of observational studies further divided according to observational model
    MATCH (:ClinicalTrialsEntry)-->(:protocolSection)-->(studyDesign:designModule)
          -->(designInformation:designInfo)
    WHERE studyDesign.studyType = 'OBSERVATIONAL'
    RETURN studyDesign.studyType as StudyType,
           designInformation.timePerspective as Perspective,
           designInformation.observationalModel as ObservationModel,
           count(studyDesign.studyType) as NoOfStudies
```

Listing S4 Querying the distribution of study types. The first query returns the number of studies by study type (interventional vs. observational). The second query focuses on interventional studies, returning the study type, allocation method (randomised vs. non-randomised), and count of studies. The third query further divides interventional studies by intervention model in addition to allocation and study type. The fourth query examines observational studies, returning study type, time perspective (prospective, retrospective, or cross-sectional), and study counts. The fifth query further categorizes observational studies by observational model along with time perspective and study type, returning the count of studies for each combination. The queries were used in creating the sunburst charts of study type distribution.

## Revealing disparities in the distribution of academia and industry studies

The Cypher query used in the analysis of study sponsorship to identify industry specific study sponsors is shown in Listing S5.

```
    MATCH  (study:ClinicalTrialsEntry)-->(:protocolSection)-->
           (:sponsorCollaboratorsModule)-->(sponsor:leadSponsor)
    WHERE  sponsor.class = 'INDUSTRY'
    RETURN sponsor.name AS Sponsor, count(study) as NoOfStudies
    ORDER BY NoOfStudies DESC
```

Listing 2 Querying for industry-sponsored studies. The query matches studies linked to their lead sponsors. It filters for sponsors from industry and returns the sponsor name (Sponsor) along with the total number of studies (NoOfStudies) associated with each sponsor, ordered by descending study count.

## The patient’s perspective: Identifying relevant studies through eligibility criteria

The results of a Cypher query showing diabetes study locations with their distance to Greifswald, can be seen in Table S1.

| **City** | **Title** | **km** |
| --- | --- | --- |
| Falkensee | Efficacy, Safety, and Tolerability of Once Daily Oral Administration of AZD5004 Versus Placebo for 26 Weeks in Adults With Type 2 Diabetes Mellitus. | 171.08 |
| Berlin | Cohort Study on Plant-based Diets (COPLANT Study) | 173.91 |
| Berlin | Effects of Almonds in Glucose-intolerant Adults (AGAMEMNON) | 173.91 |
| Berlin | Nutraceutical Improvement of Glucose Metabolism, NAFLD and Insulin Resistance by Oat-fiber Supplementation in Type 2 Diabetes Mellitus Patients | 173.91 |
| Berlin | Whey or Casein - Liver Fat Reduction and Metabolic Improvement by Fast vs. Slow Proteins | 173.91 |
| Hamburg | Smart MDI Study (CIP343) | 229.02 |
| Hamburg | Efficacy, Safety, and Tolerability of Once Daily Oral Administration of AZD5004 Versus Placebo for 26 Weeks in Adults With Type 2 Diabetes Mellitus. | 229.02 |
| Hannover | Smart MDI Study (CIP343) | 309.69 |
| Hanover | Better Control in Pediatric and Adolescent diabeteS: Working to crEate CEnTers of Reference (SWEET- Registry) | 309.69 |
| Leipzig | Smart MDI Study (CIP343) | 313.44 |
| Oldenburg | Efficacy, Safety, and Tolerability of Once Daily Oral Administration of AZD5004 Versus Placebo for 26 Weeks in Adults With Type 2 Diabetes Mellitus. | 357.66 |
| Bad Oeynhausen | Atrial Fibrillation and Diabetes Mellitus | 370.89 |
| Jena | Cohort Study on Plant-based Diets (COPLANT Study) | 372.10 |
| Jena | Treatment Satisfaction and Treatment Adherence of Diabetic Women Through the Use of FGM | 372.10 |
| Muenster | Efficacy, Safety, and Tolerability of Once Daily Oral Administration of AZD5004 Versus Placebo for 26 Weeks in Adults With Type 2 Diabetes Mellitus. | 452.56 |
| Gießen | Cohort Study on Plant-based Diets (COPLANT Study) | 504.43 |
| Essen | Efficacy, Safety, and Tolerability of Once Daily Oral Administration of AZD5004 Versus Placebo for 26 Weeks in Adults With Type 2 Diabetes Mellitus. | 519.64 |
| Düsseldorf | Study on Pancreatic Islets in Patients Undergoing (Partial) Pancreatectomy (PRECISE) | 548.58 |
| Düsseldorf | Early Percutaneous Transluminal Angioplasty in Diabetic Foot Syndrome (PTA-DFS) | 548.58 |
| Düsseldorf | The Effect of Bariatric Surgery on Insulin Sensitivity and Energy Metabolism | 548.58 |
| Cologne | Dietary Interventions During Living Kidney Donations | 559.75 |
| Bonn | Cohort Study on Plant-based Diets (COPLANT Study) | 567.15 |
| Bad Mergentheim | MyDiaMate For Remission Of Diabetes Distress In Type 1 Diabetes (MyREMEDY) | 568.71 |
| Bad Mergentheim | Precision Mental Health in Diabetes - Subtypes of Mental Health, Trajectories, and Patterns With Glycaemic Control | 568.71 |
| Bad Mergentheim | Precision Mental Health in Diabetes - Subtypes of Mental Health, Trajectories, and Patterns With Glycaemic Control | 568.71 |
| Regensburg | Cohort Study on Plant-based Diets (COPLANT Study) | 571.56 |
| Mainz | Global Research Initiative for Patients Screening on MASH | 575.16 |
| Heidelberg | Cohort Study on Plant-based Diets (COPLANT Study) | 613.12 |
| Heidelberg | Total pancrEaTectomy vs High-Risk Pancreatic anastomosiS | 613.12 |
| Heidelberg | Heidelberg Study on Diabetes and Complications | 613.12 |
| Mannheim | Efficacy, Safety, and Tolerability of Once Daily Oral Administration of AZD5004 Versus Placebo for 26 Weeks in Adults With Type 2 Diabetes Mellitus. | 613.65 |
| Wangen | Efficacy, Safety, and Tolerability of Once Daily Oral Administration of AZD5004 Versus Placebo for 26 Weeks in Adults With Type 2 Diabetes Mellitus. | 651.36 |
| Karlsruhe | Cohort Study on Plant-based Diets (COPLANT Study) | 661.95 |
| Homburg | Global Research Initiative for Patients Screening on MASH | 674.18 |
| Munich | Total pancrEaTectomy vs High-Risk Pancreatic anastomosiS | 674.26 |
| Munich | Total pancrEaTectomy vs High-Risk Pancreatic anastomosiS | 674.26 |
| Munich | Markers in Acute Pancreatitis-1 | 674.26 |
| Ulm | Whey Protein Pre-load and Postprandial Glycemia in Pregnancy | 675.91 |
| Sankt Ingbert | Efficacy, Safety, and Tolerability of Once Daily Oral Administration of AZD5004 Versus Placebo for 26 Weeks in Adults With Type 2 Diabetes Mellitus. | 688.14 |
| Tübingen | Efficacy of Whole-body Electromyostimulation (WB-EMS) Training in Participants With Pre-diabetes | 688.79 |
| Tübingen | The Influence of Genetic Variations in ELAPOR1 or ELAPOR2 on Insulin Secretion and Glucose Regulation in Humans | 688.79 |
| Freiburg | Closed-loop in Adults with Type 2 Diabetes | 780.52 |

Table S1 Diabetes studies with distance to Greifswald. The table shows study locations and titles of recruiting diabetes studies in Germany, ranked by distance to a reference location (Greifswald).

# Contrast key scenarios with the native interfaces

In the following, we provide a comparison of our use cases with what can and cannot be done via the native ClinicalTrials.gov and MDM Portal interfaces.

## Key scenarios

The following use cases from our paper highlight the key differences between what can be done in the BRAinS-Graph and contrast it against what cannot be done in the native interfaces of the data sources.

### The patient’s perspective: Identifying relevant studies through eligibility criteria

In ClinicalTrials.gov, users can search for studies that investigate a specific disease (compare (1)) and have a “recruiting” study status (compare (2)). In addition, users can enter a location (compare (3)). In our use case, we display the results of this query, namely study centers, with the study center’s distance to a reference location. In the native ClinicalTrials.gov interface, this distance is not displayed. However, in the search interface, a “radius bar” can be used “to pick the number of miles you are willing to travel”^[9]^.

Our use case also includes querying the graph for specific inclusion and exclusion criteria (inclusion criteria “diabetes”, exclusion criteria “pregnancy”, compare Listing 3 and Figure 8). The MDM Portal contains structured eligibility criteria. However, a search for specific inclusion and exclusion criteria of a study is currently not possible. The UMLS codes used for semantic annotation of study forms cannot be directly queried in the MDM Portal. Our graph, however, enables both searching for eligibility criteria as well as querying the UMLS codes of the MDM Portal’s study forms. In ClinicalTrials.gov, specific querying of inclusion and exclusion criteria is not possible. In general, querying of eligibility criteria is possible beyond standard filters, such as age, sex, acceptance of healthy volunteers, using the Expert Search and the search area “EligibilityCriteria”. However, the search cannot distinguish/specify if inclusion or exclusion criteria.

### Analyzing study information

In this use case, we analyzed the annotation accuracy of studies from ClinicalTrials.gov. We queried the set of MeSH terms used for annotation of studies. This analysis is not directly possible in the native ClinicalTrials.gov interface. A workaround would be to download study results as json files which include the mesh terms used for annotation. These files could then be analyzed in a post-processing step, after the download and outside of the ClinicalTrials.gov interface.

## Other scenarios

The following comparisons contrast the differences in what can and cannot be done in the native interfaces for all other use cases.

### Statistical analyses

#### Study type distribution for meta-analysis

The extraction of information needed to create the sunburst charts of study type distribution (compare Figure 5) is possible with the search areas of the Expert Search in ClinicalTrials.gov. Search results can be downloaded as json files that contain all necessary data fields.

#### Regional and socio-economic distribution of study centers in Germany

The information needed to create the geographic map displaying the number of studies per city (compare Figure 6, Geographic distribution of actively recruiting studies in Germany) can be extracted from ClinicalTrials.gov. However, this information would need an additional processing step to sum up studies per city. In our graph, we can directly extract the number of studies per geolocation/city. The Map View^[10]^ in ClinicalTrials.gov shows the number of studies per region or country. In Europe, this number is per country and can therefore not be shown in more detail for a single country, e.g. for Germany as in our use case.

#### Revealing disparities in the distribution of academia and industry studies

It is possible to query the number of studies with a lead sponsor from industry or with at least one industry collaborator in ClinicalTrials.gov using the Expert Search and the “CollaboratorClass” search area (by entering the query “AREA[CollaboratorClass] INDUSTRY” or “AREA[LeadSponsorClass] INDUSTRY”).

The search results can be displayed in different views, one option is called “By Topic”: “By Topic shows studies from your search in a table organized by topic. This feature is still under development. Currently, the search results can only be organized by the Sponsor/Collaborator.”^[11]^ This limitation does not exist in our graph. However, since the search results in ClinicalTrials.gov can be organized by sponsor/collaborator, it is possible to check for the top industry sponsors by number of sponsored studies in the native interface (compare Table 2, Top industry sponsors).

However, in general, more sophisticated counts are not possible directly in the native ClinicalTrials.gov interface. It is not possible to count multiple factors at the same time in one query, e.g. the top industry sponsors with the number of sponsored interventional and the number of sponsored observational studies.

# How to

Our framework can be used to build the knowledge base on a user’s local machine. Once the user has downloaded the repository, the following steps must be undertaken: First, the user must download all of the data files, which are to be integrated into the knowledge base. The user is required to store the data files in a folder that must be specified when the program is run. Secondly, the user must specify the connection parameters for their running graph database (Neo4j instance) in the configuration file. A template of the configuration file is provided for reference. It is essential that the database is running and accessible. Thirdly, the user must execute the python scripts. This will result in the population of the graph database and the creation of the knowledge base. For more detailed instructions, we direct the reader to the README file provided in our repository (<https://git.uni-greifswald.de/MILA_public/BRAinS-Graph>).

# Technical requirements

All imports and analyses were conducted on a laptop with the following specifications:

- Hardware: HP EliteBook 830 G6
- OS: Debian Bookworm (LMDE 6 (faye) x86_64)
- CPU: Intel i5 with 8 cores, 3.9GHz
- Memory: 32 GB RAM

Indicative runtimes:

- ETL process runtime:
  - 1319 json files from ClinicalTrials.gov imported with study2neo4j: approx. 36 hours
  - 1760 xml files imported with mdm2neo4j: approx. 2 hours
- Query runtimes:
  - Listing 2 (Querying for recruiting diabetes studies in Germany): *Started streaming 44 records after 75 ms and completed after 245 ms*.
  - Listing 3 (Querying for eligibility criteria): *Started streaming 4 records after 44 ms and completed after 77 ms*.
  - Listing 4 (Querying for type 1 diabetes studies): *Started streaming 1 records after 20 ms and completed after 325 ms*.
  - Listing 5 (Type 1 diabetes studies without the corresponding MeSH annotation): *Started streaming 8 records after 34 ms and completed after 91 ms*.

Database configuration:

- neo4j.conf: dbms.security.procedures.unrestricted=apoc.* (to enable apoc procedures), dbms.security.allow_csv_import_from_file_urls=true (to allow file imports); dbms.memory.heap.initial_size=4G, dbms.memory.heap.max_size=8G, dbms.memory.pagecache.size=2G (to increase memory allocation for large imports)
- apoc.conf: apoc.import.file.enabled=true

For more (technical) information about Neo4j, we direct the reader to the respective documentation^[12]^ and Neo4j’s Operation Manual^[13]^.

# Validation on a new data set

We validated the BRAinS-Graph framework on a second data set. The data set includes new data from the MDM Portal and ClinicalTrials.gov. More precisely, it includes all eligibility criteria from the MDM Portal, without restriction to a specific disease, and a set of “prosthetics” studies from ClinicalTrials.gov without restriction to a specific region.

## Data selection and import

MDM Portal search query (on February 26th, 2026): search?keywords=15492&keywords=4410

The search returns 9,573 medical data models. In the MDM Portal, keywords can be assigned to all forms. TOC entries have been generated for the overview on the website. These are stored in the MDM Portal database in a way that specifies which keywords are to be used. For TOC 3, for example, keyword 4410 is specified for use in the search. Keyword 4410 comes from the mesh15 dataset and has the translation ‘Eligibility Determination’ stored in the database. The other keyword for clinical trials (15492) also originates from the mesh15 dataset.

ClinicalTrials.gov search query (on March 16th, 2026): prosthetics

The search returns 237 studies. We used the Expert Search of ClinicalTrials.gov. We downloaded the results as individual json files with all available Data Fields.

For demonstration purposes, we used a random subset of 5,500 xml files from the medical data models described above and the 237 json files from the ClinicalTrials.gov “prosthetics” dataset.

## Knowledge graph

The resulting graph contains 438,994 nodes and 985,344 relationships. In addition, we integrated a random subset of the ClinicalTrials.gov json files from the initial diabetes/Germany dataset of February 2025. This subset contains 1,255 studies. In total, we were able to link 63 distinct ClinicalTrials.gov studies to 63 medical data models from the MDM Portal, mapping each study to a unique medical data model. This improves the numbers from our initial diabetes knowledge graph. Examples for the matched studies and their eligibility criteria forms can be seen in Figure S3. Screenshot of Neo4j Browser. The figure shows two queries and their results. The queries show the number of matches between studies and medical data models and examples of these matches.

Figure S3 Screenshot of Neo4j Browser. The figure shows two queries and their results. The queries show the number of matches between studies and medical data models and examples of these matches.

# References

[1] Pigeot I et al. Making epidemiological and clinical studies FAIR using the example of COVID-19. Datenbank-Spektrum. 2024;24:117–128. doi: 10.1007/s13222-024-00477-2
[2] Zarin DA, Tse T, Williams RJ, Califf RM, Ide NC. The ClinicalTrials.gov results database–update and key issues. New England Journal of Medicine. 2011;364:852–860. doi: 10.1056/NEJMsa1012065
[3] Hasselblatt H et al. The German Clinical Trials Register: challenges and chances of implementing a bilingual registry. Journal of Evidence-Based Medicine. 2009;2(1):36-40. doi: 10.1111/j.1756-5391.2009.01001.x
[4] Hoffmann J et al. The DZHK research platform: maximisation of scientific value by enabling access to health data and biological samples collected in cardiovascular clinical studies. Clinical Research in Cardiology. 2023;112(7):923–941. doi: 10.1007/s00392-023-02177-5
[5] Dugas M et al. Portal of medical data models: information infrastructure for medical research and healthcare. Database. 2016;bav121. doi: 10.1093/database/bav121
[6] Riepenhausen S et al. Europe’s largest research infrastructure for curated medical data models with semantic annotations. Methods of Information in Medicine. 2024;63:52–61. doi: 10.1055/s-0044-1786839
[7] Bodenreider O. The Unified Medical Language System (UMLS): integrating biomedical terminology. Nucleic Acids Research. 2004;32:D267–D270. doi: 10.1093/nar/gkh061
[8] Hegselmann S et al. Pragmatic MDR: a metadata repository with bottom-up standardization of medical metadata through reuse. BMC Medical Informatics and Decision Making. 2021;21:160. doi: 10.1186/s12911-021-01524-8

1. <https://www.who.int/clinical-trials-registry-platform>, accessed Dec 9, 2024
2. <https://www.who.int/clinical-trials-registry-platform/network/who-data-set>, accessed Dec 3, 2024
3. <https://www.bfarm.de/EN/BfArM/Tasks/German-Clinical-Trials-Register/_node.html>, accessed Dec 9, 2024
4. <https://www.clinicaltrialsregister.eu/>, accessed Dec 9, 2024
5. <https://euclinicaltrials.eu/>, accessed Dec 9, 2024
6. <https://crmdr.ecrin.org/>, accessed Dec 9, 2024
7. <https://www.isrctn.com/>, accessed Dec 9, 2024
8. <https://www.cdisc.org/standards/data-exchange/odm>, accessed Aug 22, 2025
9. https://clinicaltrials.gov/find-studies/how-to-search#a3, last accessed Mar 25, 2026
10. https://clinicaltrials.gov/find-studies/how-to-use-search-results#map-view, last accessed Mar 25, 2026
11. https://clinicaltrials.gov/find-studies/how-to-use-search-results#by-topic-view, last accessed Mar 25, 2026
12. https://neo4j.com/docs/
13. https://neo4j.com/docs/operations-manual/current/
